# Cysteinylprolyl imide (CPI) peptide: a highly reactive and easily accessible crypto-thioester for chemical protein synthesis†
†Electronic supplementary information (ESI) available. See DOI: 10.1039/c9sc00646j


**DOI:** 10.1039/c9sc00646j

**Published:** 2019-05-09

**Authors:** Masafumi Yanase, Koki Nakatsu, Charlane Joy Cardos, Yoshiki Konda, Gosuke Hayashi, Akimitsu Okamoto

**Affiliations:** a Department of Chemistry and Biotechnology , The University of Tokyo , 7-3-1 Hongo, Bunkyo-ku , Tokyo 113-8656 , Japan . Email: okamoto@chembio.t.u-tokyo.ac.jp; b Department of Biomolecular Engineering , Graduate School of Engineering , Nagoya University , Furo-cho, Chikusa-ku , Nagoya 464-8603 , Japan . Email: hayashi@chembio.nagoya-u.ac.jp; c Research Center for Advanced Science and Technology , The University of Tokyo , 4-6-1 Komaba, Meguro-ku , Tokyo 153-8904 , Japan

## Abstract

Native chemical ligation (NCL) between the C-terminal peptide thioester and the N-terminal cysteinyl-peptide revolutionized the field of chemical protein synthesis. The difficulty of direct synthesis of the peptide thioester in the Fmoc method has prompted the development of crypto-thioesters that can be efficiently converted into thioesters. Cysteinylprolyl ester (CPE), which is an *N*–*S* acyl shift-driven crypto-thioester that relies on an intramolecular *O*–*N* acyl shift to displace the amide-thioester equilibrium, enabled *trans*-thioesterification and subsequent NCL in one pot. However, the utility of CPE is limited because of the moderate thioesterification rates and the synthetic complexity introduced by the ester group. Herein, we develop a new crypto-thioester, cysteinylprolyl imide (CPI), which replaces the alcohol leaving group of CPE with other leaving groups such as benzimidazolidinone, oxazolidinone, and pyrrolidinone. CPI peptides were efficiently synthesized by using standard Fmoc solid-phase peptide synthesis (SPPS) and subsequent on-resin imide formation. Screening of the several imide structures indicated that methyloxazolidinone-*t*-leucine (MeOxd-Tle) showed faster conversion into thioester and higher stability against hydrolysis under NCL conditions. Finally, by using CPMeOxd-Tle peptides, we demonstrated the chemical synthesis of affibody *via* N-to-C sequential, three-segment ligation and histone H2A.Z *via* convergent four-segment ligation. This facile and straightforward method is expected to be broadly applicable to chemical protein synthesis.

## Abstract 2

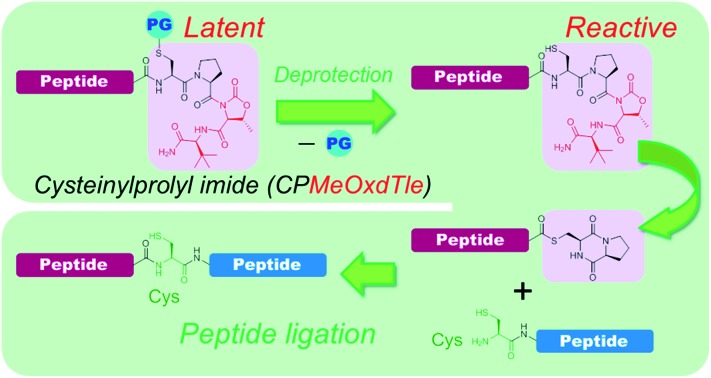
A new crypto-thioester, cysteinylprolyl imide (CPI) peptide, offers a practical synthetic pathway and reliable reaction rate to be successfully applied to chemical protein synthesis.

## Introduction

Native chemical ligation (NCL)^1^ between N-terminal cysteinyl peptides and C-terminal α-thioester peptides is an indispensable technique in total chemical protein synthesis.^2^ Given that the direct synthesis of peptide α-thioesters in Fmoc-based solid-phase peptide synthesis (SPPS) is not straightforward because of the instability of the thioester moiety during the repeated Fmoc-deprotection steps, a large number of thioester precursors that can be efficiently converted into thioesters have been developed to date.^3^–^7^ While Dawson's diaminobenzoyl linker^4^ and Liu's hydrazide^5^ have become popular, intramolecular acyl shift-based thioester precursors are promising because they can be transformed into thioesters under NCL conditions.

In this context, a number of *N*–*S* acyl-shift-type thioester precursors have been reported^6^,^7^ in which *N*–*S* acyl shift from an amino group to a β- or γ-mercapto group is induced and subsequent exposure to an excess of external thiol in the solution leads to *trans*-thioesterification. Given that the initial *N*–*S* acyl shift normally proceeds under acidic conditions, most of these *N*–*S* acyl shift type thioester precursors are less likely to generate reactive thioesters at the neutral pH used in peptide ligation. Nevertheless, several *N*–*S* acyl shift type thioester precursors, called crypto-thioesters, can be utilized to operate one-pot thioester formation and NCL.7d,k,l,o,p,r–x One of the advantages of *N*–*S* acyl shift-driven crypto-thioesters is that their activity can be easily suppressed by protecting the mercapto group and they can be used for N-to-C sequential assembly.^8^ However, for many crypto-thioesters, the monomer unit with the mercapto group being protected by some acid-stable protecting group is not commercially available. As an exception, the 2-hydroxy-5-nitrobenzyl cysteine (Hnb–Cys), developed by Aucagne,7*u* overcomes these problems because Hnb–Cys is synthesized from the cysteine (Cys) through subsequent introduction of the Hnb unit by reductive amination at the amino group of cysteine. The use of Cys makes synthetic access to a broad range of mercapto-protected Hnb–Cys derivatives straightforward. Furthermore, the Hnb group plays a role as an acid catalyst for the *N*–*S* acyl shift, and thus NCL rates with the Hnb–Cys peptide are relatively fast. However, ligation of three or more peptide segments with the Hnb–Cys peptide in the N-to-C direction has not been reported to date.

**Scheme 1:**
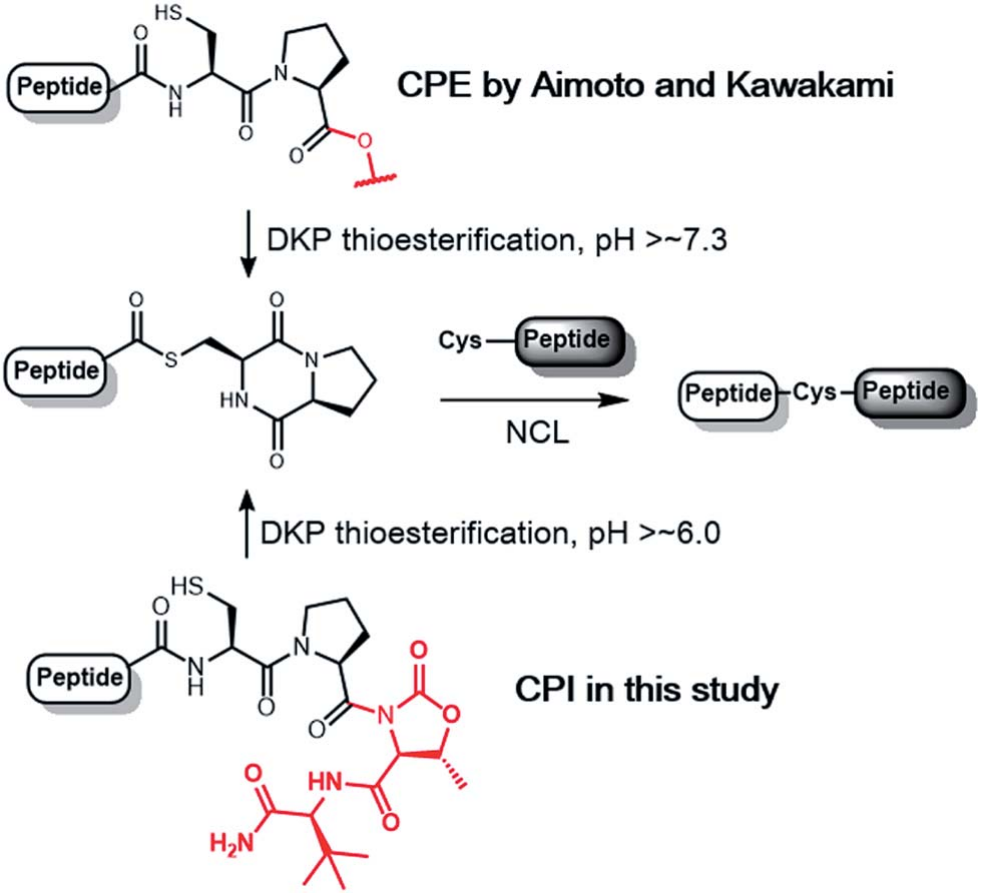
Thioesterification of CPE and CPI to the diketopiperazine (DKP) form.

Among *N*–*S* acyl shift-driven crypto-thioesters, cysteinylprolyl ester (CPE) relies on an elegant intramolecular *O*–*N* acyl shift to displace the amide-thioester equilibrium and form the diketopiperazine (DKP) thioester under NCL conditions (Scheme 1).7d,r Because of the unique pathway, the thioester formation of CPE is favored at higher pH values. Nevertheless, the rates of thioesterification at neutral and lower pH are relatively slow. In addition, CPE has an increased synthetic complexity in that the Xaa–Cys dipeptide is required to prevent dipeptide deletion, making it difficult to introduce protecting groups of Cys in CPE.

We herein report the construction of cysteinylprolyl imide (CPI) peptides in which the ester moiety in CPE was replaced with tertiary imides (Scheme 1). The latter can be generated on resin after peptide elongation. The CPI synthesis does not require Xaa–Cys dipeptide coupling during peptide elongation, providing flexibility to introduce various Cys derivatives with different protecting groups. CPI peptides spontaneously converted into the thioester under NCL conditions and showed faster rates of thioesterification than CPE peptides. The utility of the CPI peptides was demonstrated by the chemical syntheses of two proteins, affibody and histone H2A.Z through N-to-C ligation and convergent synthesis, respectively.

## Results and discussion

### Design and syntheses of CPI peptides

**Scheme 2:**
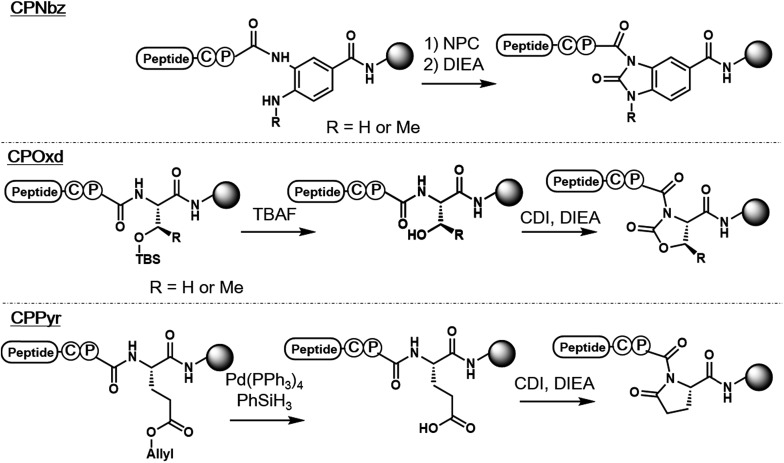
Syntheses of CPI peptides used in this report.

In the pursuit of a more generally applicable crypto-thioester preparation method that uses commercially available reagents, we focused on three on-resin activatable leaving groups, namely, Nbz,4*a* oxazolidinone (Oxd)^9^ and pyrrolidinone (Pyr),^10^ which can be synthesized from 3,4-diaminobenzoic acid (Dbz), serine, and glutamate, respectively, after peptide elongation (Scheme 2). We envisaged that by introducing these leaving groups next to Cys–Pro, these peptides would work as a crypto-thioester *via* the same reaction pathway as CPE. First, model peptides containing CPNbz **1**, CPOxd **2G**, and CPPyr **3** with a *t*-butyl disulfide protecting group on the Cys residue were synthesized (Table S1 and Fig. S5†). In the synthesis of peptide **1**, amino acids were elongated on Dawson Dbz AM resin followed by activation of Dbz according to previous procedures.4*a* For peptides **2G** and **3**, Fmoc–Ser(TBDMS)–OH^11^ and Fmoc–Asp(OAll)–OH^12^ were used for the orthogonal deprotection and subsequent Oxd and Pyr formation, respectively, after peptide elongation on Rink Amide resin. The TBDMS and the allyl groups were removed by tetrabutylammonium fluoride (TBAF) and tetrakis(triphenylphosphine)palladium(0), respectively. For Oxd formation, screening of activating reagents showed that carbonyldiimidazole (CDI) was the most efficient (Fig. S1†) and additives such as *N*,*N*-dimethyl-4-aminopyridine, *N*-hydroxysuccinimide, and pyridine decreased the cyclization efficiency (Fig. S2†). For Pyr formation, screening of the activators revealed that CDI can also be used to produce CPPyr most efficiently (Fig. S3†). In HPLC analyses, both CPOxd and CPPyr peptides showed broad peaks that were mainly composed of two peaks, which could be attributed to *trans* and *cis* isomers (Fig. S2, S3, S5 and S6†).^13^ Since these isomers were in equilibrium at room temperature (Fig. S6†), we used the mixture of *trans* and *cis* isomers in this study.

For control experiments, we also synthesized the CPE peptide by using Fmoc-Gly–Cys(Trt)-OH dipeptide coupling7d,r and postsynthetic introduction of a disulfide protecting group into the Cys residue. It is known that 3-nitro-2-pyridinesulfenyl-protected Cys was directly generated from trityl-protected Cys by using a TFA-based cleavage cocktail containing 2,2′-dithiobis(5-nitropyridine).^14^ By using diethyl disulfide instead of nitropyridyl disulfide, we obtained ethylsulfenyl-protected CPE peptide **4** (Table S1, Fig. S4 and S5†), while the *t*-butylsulfenyl-protected CPE peptide was not generated in the cleavage cocktail containing di-*t*-butyl disulfide (Fig. S4†).

### MESNa thioesterification of CPE and CPI peptides

**Fig. 1:**
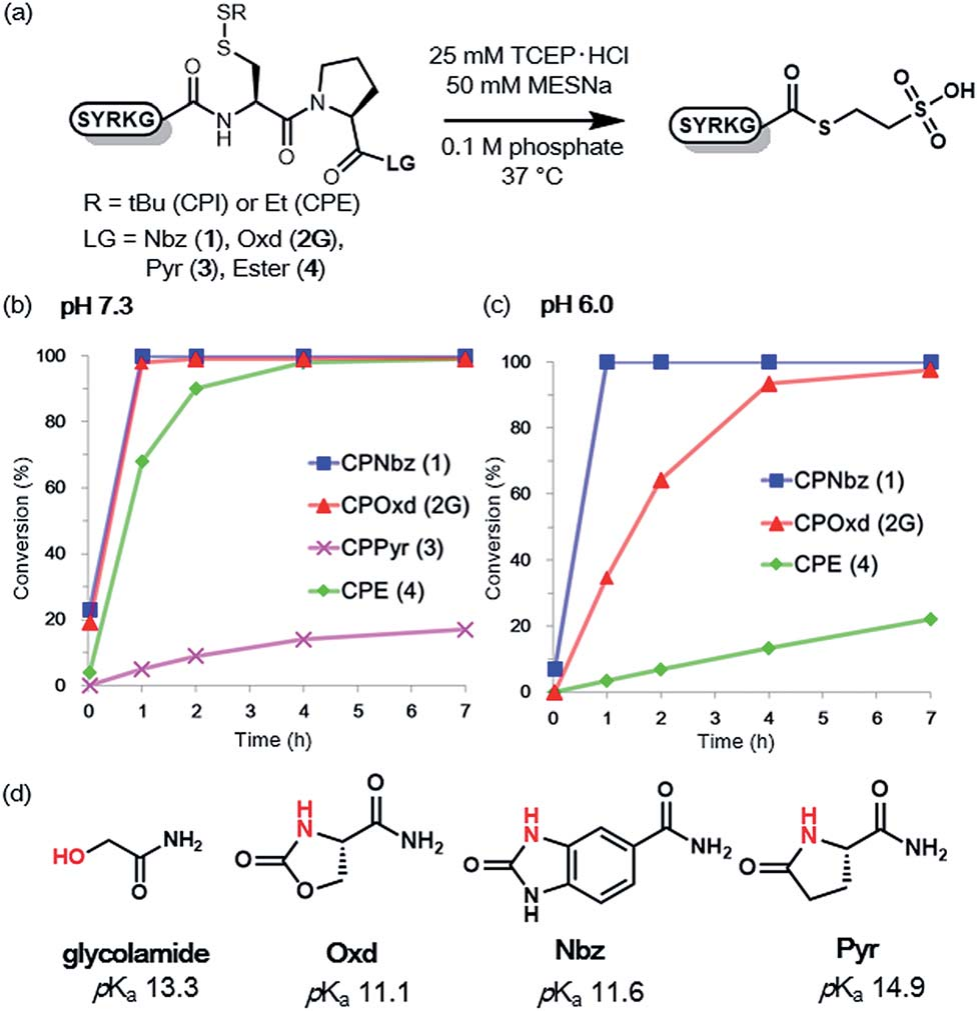
MESNa thioester formation of CPI peptides and CPE peptides. (a) Reaction scheme of MESNa thioesterification. Time course analysis of MESNa thioesterification of CPI and CPE peptides at (b) pH 7.3 and (c) pH 6.0. *t* = 2 min, 1 h, 2 h, 4 h, and 7 h. (d) p*K*_a_ values calculated with Percepta (ACD Labs) for the amides and the alcohol (shown in red) used in this study.

To compare the rates of the thioester formation from CPI and CPE peptides, the peptides (**1**, **2G**, **3**, **4**, 1 mM) were treated with 2 mM sodium 2-mercaptoethane sulfonate (MESNa) in 0.1 M sodium phosphate buffer (pH 7.3 or 6.0) containing 25 mM TCEP·HCl at 37 °C (Fig. 1 and S7–S10†). At pH 7.3, the thioester conversion rates of the CPNbz **1** and CPOxd **2G** peptides were remarkably fast (Fig. 1b). For CPPyr peptide 3, the thioester formation reaction did not proceed efficiently and a desulfurized product appeared over time (Fig. S9†).^15^ Therefore, we concluded that the Pyr group is not suitable as a leaving group in the CPI peptide. At pH 6.0, CPNbz and CPOxd also rapidly transformed into the thioester, while CPE did not. The order of reactivity at pH 6.0 was CPNbz > CPOxd > CPE (Fig. 1c).

To explain this difference, the p*K*_a_ values of the alcohol moiety of glycolamide and the amino moiety of imides such as Nbz, Oxd, and Pyr were estimated by using ACD Labs software.^16^ As shown in Fig. 1d, compared with glycolamide, the p*K*_a_ values of Nbz and Oxd were 2 p*K*_a_ units lower and the p*K*_a_ of Pyr was 1.6 p*K*_a_ units higher, which is consistent with our results on the reactivity. The higher reactivity of CPNbz than CPOxd could have resulted from the rigidity of the conformation of the amide bond caused by the direct connection of the benzene ring to the imide moiety in CPNbz.

### Study of the NCL with model CPOxd peptides

To investigate the ligation efficiency of CPOxd peptides, we prepared CPOxd peptides with nine different amino acids at the ligation junction (Table 1). NCL reactions were then conducted with each peptide (1 mM) and Cys (2 mM) in the presence of 25 mM TCEP·HCl, 100 mM MPAA^17^ at 37 °C and a final pH of 7.0 (Table 1 and Fig. S11†). In all cases, ligation proceeded with excellent conversion yields (88–98%) and was complete in 2–4 h, even when peptides containing the more sterically hindered Val thioester were used (Table 1, entry 9 and Fig. S11j†). At room temperature, ligation of peptide 2G also proceeded well in 7 h (Table 1, entry 10 and Fig. S11k†). Notably, no by-products containing Cys–Pro residues were detected, suggesting that DKP formation proceeds much faster than direct thiolysis at the Oxd imide with external thiol (*i.e.* MPAA and cysteine in this case) and subsequent intramolecular *S*–*N* acyl shift in cysteine. Epimerization of the Ala residue at the junction site of peptide **2A** was also evaluated under NCL conditions by comparing the HPLC peak of d-Ala-containing peptide **2a** (Fig. S11 and S12†). Only a trace amount of epimerization (*ca.* 0.7%) was identified in the NCL mixture of **2A**, suggesting that the CPOxd peptide has characteristics similar to those of previous crypto-thioesters.7l,r,u,x,^18^

**Table 1:** Native chemical ligation between CPOxd peptides and cysteine

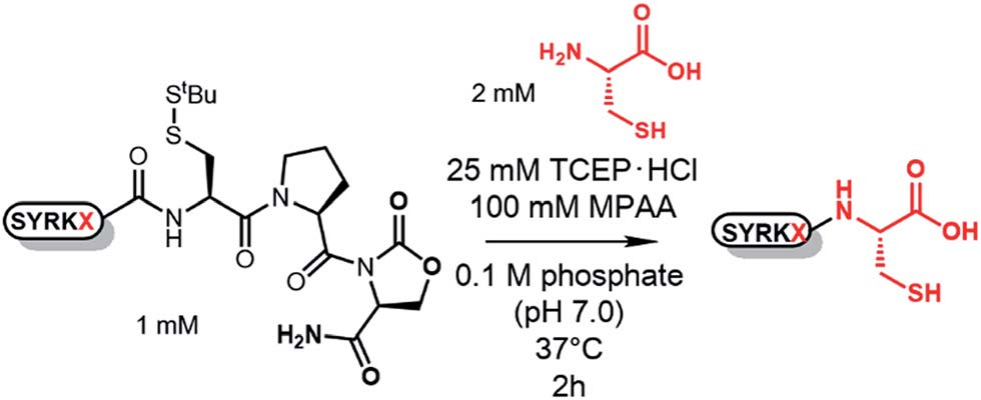			
Entry	Peptides	Xaa	Conversion (%)
1	**2G**	Gly	98
2	**2A**	Ala	95
3	**2Q**	Gln	88
4	**2F**	Phe	90
5	**2S**	Ser	94
6	**2N**	Asn	96
7	**2L**	Leu	95
8	**2K**	Lys	91
9	**2V**	Val	92^*a*^
10	**2G**	Gly	96^*b*^

^*a*^Reaction completed in 4 h.

^*b*^Reaction was conducted at room temperature and completed in 7 h.

### Optimization of imide linker stability to hydrolysis with protecting groups

**Fig. 2:**
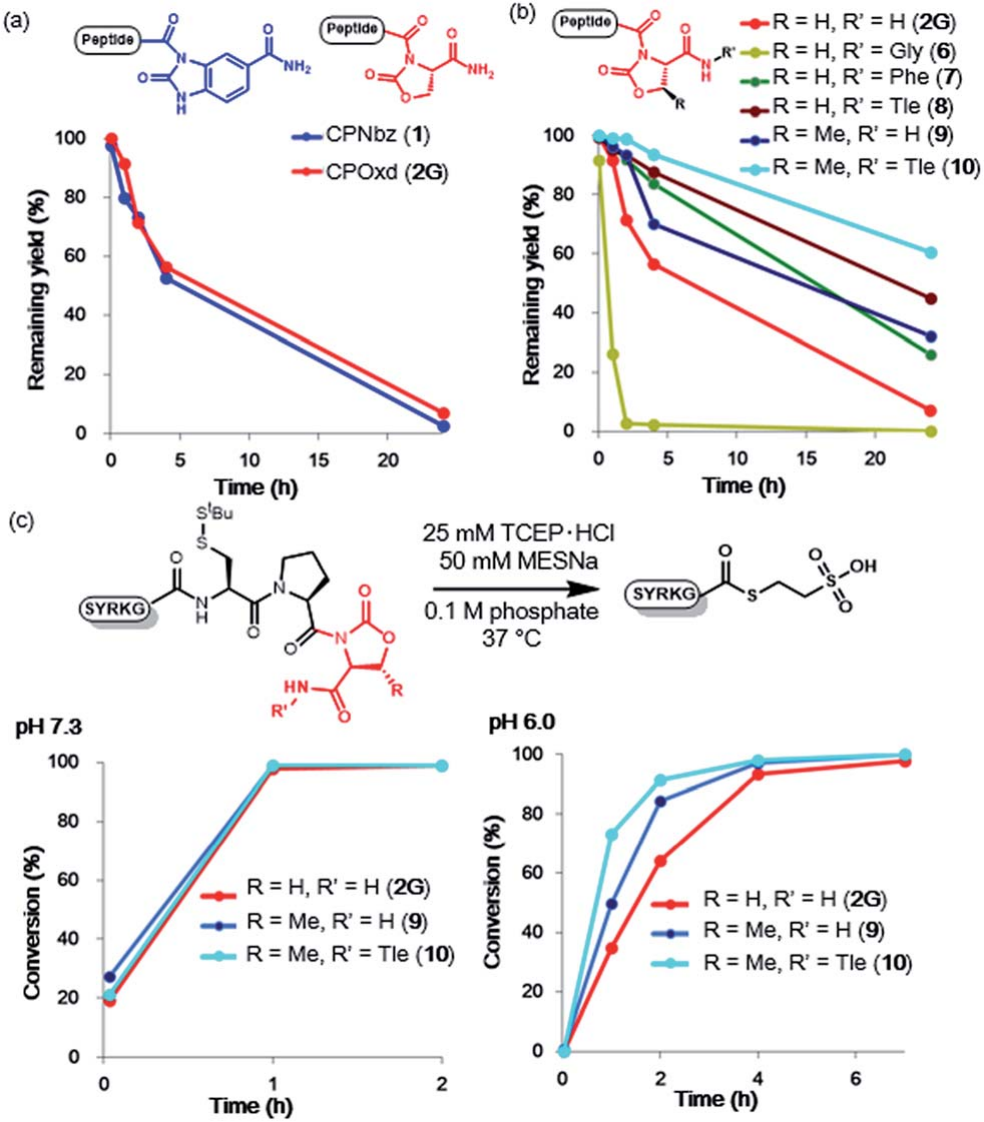
Imide optimization for resistance to hydrolysis and for thioester formation. (a) Remaining yields of CPNbz and CPOxd peptides in phosphate (pH 7.0) at 37 °C. (b) Remaining yields of Oxd-modified CPOxd peptides in phosphate (pH 7.0) at 37 °C. (c) Comparison of thioester formation of CPOxd and CPMeOxd peptides. *t* = 2 min, 1 h, 2 h, 4 h, and 7 h.

When a peptide crypto-thioester is applied to N-to-C sequential peptide ligation, the activity of the crypto-thioester has to be suppressed by introducing protecting groups to prevent self-cyclization and homodimerization of the peptide, and this latent crypto-thioester should be stable to hydrolysis.^8^ To validate the stability against hydrolysis, peptides **1** and **2G** were exposed to phosphate buffer (0.1 M, pH 7.0) without TCEP. The time course HPLC analyses indicated that, for both peptides, half of the initial amount decomposed after 4 h to generate the hydrolyzed peptide acid (Fig. 2a, S13, and S14†). Notably, CPOxd peptide **2G** generated the hydrolyzed peptide acid Cys–Pro–OH and Cys–Pro–Ser as well as the peptide amide Cys–Pro–NH_2_, presumably through an intramolecular transition from tertiary to secondary imide and subsequent hydrolysis (Fig. S14†). We hypothesized that insertion of an amino acid at the C-terminus could suppress these undesirable reactions. Therefore, three CPOxd peptides with C-terminal Gly **6**, Phe **7**, and Tle **8** were synthesized (Table S1 and Fig. S5†) and their hydrolytic stability was tested (Fig. 2b and S15b–d†). Insertion of a C-terminal amino acid with a bulky side chain drastically enhanced the hydrolytic stability, whereas the Gly-inserted CPOxd peptide decreased the stability.

Given that the hydrophobicity around the imide moiety is important for increasing resistance to hydrolysis, methyloxazolidinone (MeOxd), which is synthesized from threonine, should show higher hydrolytic stability compared with Oxd. Tests to establish the resistance of CPMeOxd peptide **9** towards hydrolysis suggested that the stability of CPMeOxd peptides was higher than that of CPOxd peptides (Fig. 2b and S15e†). Furthermore, CPMeOxd-Tle peptide **10** showed a significant hydrolytic stability, with 95% peptide remaining intact in 4 h (Fig. 2b and S15f†), and it was the most stable structure among CPI peptides tested in this study. Although we verified the influence of substituents on CPNbz as well, CPNbz–Tle peptide **11** and CPMeNbz4*c* peptide **12** did not influence the stability towards hydrolysis (Fig. S16†).

### Thioester formation and NCL reaction of optimized CPMeOxd-Tle peptides

We evaluated the thioester formation for the CPMeOxd peptides **9** and **10** under the conditions detailed in Fig. 1. Interestingly, both a methyl group on the oxazolidinone ring and Tle at the C-terminus enhanced the rate of thioesterification (Fig. 2c and S17†). A previous study in which insertion of an amino acid to the C-terminus of the CPE peptide, especially an amino acid with a bulky side chain, accelerated the thioester formation is consistent with our results.7*r*

For comparison of kinetics with other *N*–*S* acyl shift-based crypto-thioesters, we conducted NCL kinetics analysis under reaction conditions similar to those used for Hnb–Cys,7*u* which is one of the *N*–*S* acyl shift-based crypto-thioesters with faster NCL rates (Fig. S18†). The apparent second-order rate constant of the Ala–CPMeOxd–Tle peptide **13** was 1.1 M^–1^ s^–1^ at 37 °C (Table S1 and Fig. S18c–e†), while that of the Ala–HnbCys peptide was reported to be 0.048 M^–1^ s^–1^ at 37 °C.7*u* Although the peptide sequence and the ratio of the peptide substrate are different, and *t*-butylthio-protected Hnb–Cys was used in the NCL reaction in a previous study,7*u* our CPMeOxd–Tle has faster or comparable NCL rates compared with the Hnb–Cys crypto-thioester. Moreover, we calculated the apparent second-order rate constant for NCL with the peptide alkyl thioester (MESNa) **5A** to be 2.4 M^–1^ s^–1^ (Table S1 and Fig. S18c, d, and f†). Therefore, CPMeOxd–Tle has 2.2-fold slower NCL rates than the alkyl thioester. On the other hand, according to a previous report, SEA, another *N*–*S* acyl shift-based crypto-thioester with faster kinetics, has 8.5-fold slower NCL rates than the alkylthioester (MPA)^19^ (Fig. S18b†). Therefore, it can be concluded that CPMeOxd–Tle has comparable or faster NCL rates compared with the other *N*–*S* acyl shift-based crypto-thioesters.

### Comparison of the CPMeOxd–Tle peptide with the hydrazide peptide

To compare the properties of our CPI peptides with those of the hydrazide peptide,^5^ which is currently one of the most popular thioester precursors, we evaluated the yields of SPPS, NCL rates, and yields of NCL with a cysteine using each peptide with the same amino acid sequence (Fig. S19a and S20a†). The yields of each peptide synthesis, which were calculated based on Fmoc-quantification of Fmoc–Gly attached resin, were 43% for the CPMeOxd–Tle peptide and 63% for the hydrazide peptide. In NCL experiments, while the hydrazide peptide requires azidation at –15 °C for 15 min before an N-cysteinyl peptide in the NCL buffer is added and the pH is adjusted with an aqueous NaOH solution, the CPI peptides can be used simply by mixing with an N-cysteinyl peptide in the NCL buffer. The HPLC monitoring of NCL time course indicated that both CPMeOxd–Tle and hydrazide peptides were completely consumed within 10 min and the Cys-ligated products appeared (Fig. S19 and S20†). The isolated yields were reasonable; 76% and 58% for the CPMeOxd–Tle and hydrazide peptides, respectively. Therefore, we concluded that the NCL rate of CPI peptides could be comparable with that of hydrazide peptides.

One possible advantage of the *N*–*S* acyl shift based crypto-thioester toward other thioester precursors is its applicability to one-pot N-to-C ligation of multiple peptide segments.8b,c When the thiol in the CPI peptide is protected, the thioesterification is completely suppressed. Once the protecting group of the thiol is removed, the crypto-thioester is activated. Due to the *in situ* formation of the active thioester under the NCL condition, CPI peptides would be useful especially in the synthesis of larger proteins.

### Synthetic applications

**Fig. 3:**
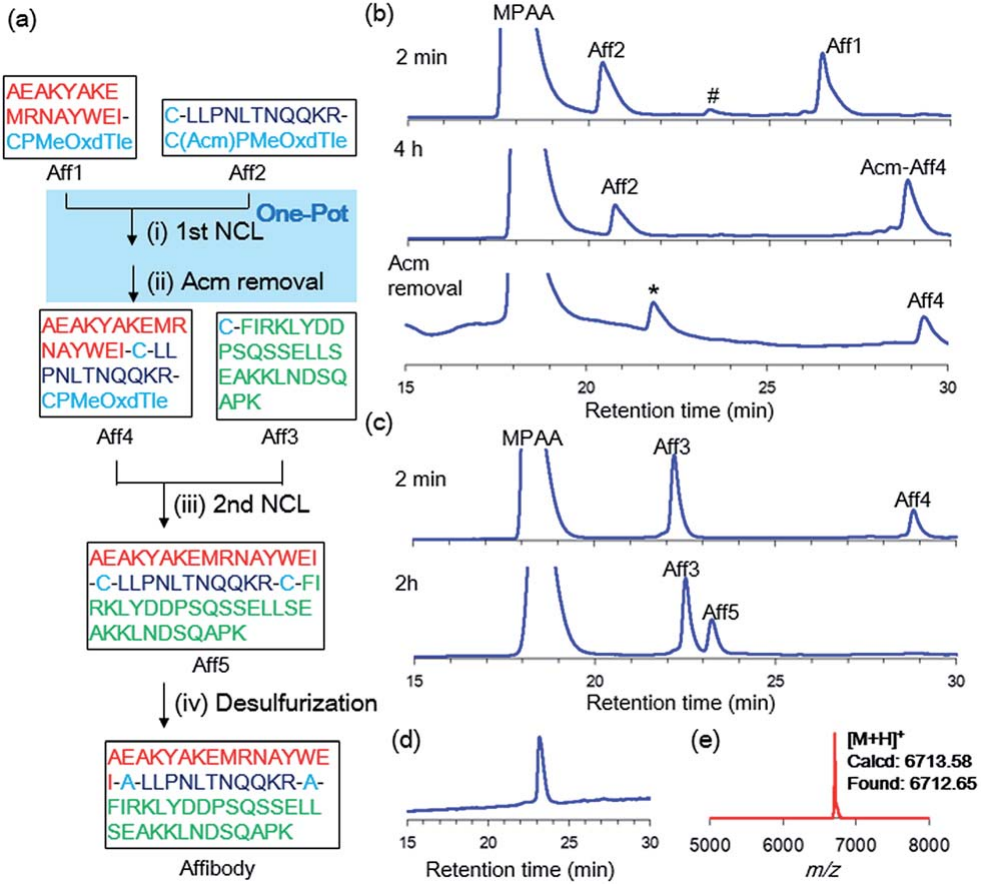
Synthesis of affibody by using CPI peptides. (a) Synthetic route. (i) 25 mM TCEP·HCl, 100 mM MPAA, 6 M Gdn·HCl, 0.2 M sodium phosphate (pH 7.0), 37 °C, 4 h. (ii) 40 equiv. PdCl_2_, room temperature, 40 min, 54%. (iii) 25 mM TCEP·HCl, 100 mM MPAA, 6 M Gdn·HCl, 0.2 M sodium phosphate (pH 7.0) 37 °C, 2.5 h, 53%. (iv) 500 mM TCEP·HCl, 500 mM glutathione, 150 mM VA-044, 6 M Gdn·HCl, 0.2 M sodium phosphate (pH 7.0), 37 °C, 1 h, 82%. (b) The first ligation between **Aff1** and **Aff2**. # = **Aff1**-diketopiperazine thioester. * = Acm-deprotected **Aff2**. (c) The second ligation between **Aff4** and **Aff3**. (d) Analytical HPLC trace and (e) MALDI-MS of purified Affibody.

Our new crypto-thioester was applied to the chemical synthesis of two kinds of proteins, ZHER2 affibody and histone H2A.Z. ZHER2 affibody consists of 58 amino acids and was developed to target the HER2 receptor.^20^ To test the applicability of CPMeOxd–Tle peptides, we synthesized the 58 amino-acid protein through sequential three-segment N-to-C ligation, in which the latent state of the crypto-thioester (*i.e. S*-protected state) should be stable during both the first ligation and subsequent deprotection (Fig. 3a). Ala residues at positions 17 and 29 were replaced with Cys to split the protein into three peptide segments: **Aff1** (1–16), **Aff2** (17–28) and **Aff3** (29–58). The C-terminus of **Aff1** was connected to a reactive crypto-thioester CPMeOxd–Tle and the C-terminus of **Aff2** was connected to a latent crypto-thioester C(Acm)PMeOxd–Tle (Fig. 3a). All three segments were synthesized by using Fmoc–SPPS (Fig. S18†). The first ligation between **Aff1** (2.0 mM final conc) and **Aff2** (2.5 mM final conc) was conducted in NCL buffer (6 M Gdn·HCl, 0.2 M sodium phosphate (pH 7.0), 100 mM MPAA, 25 mM TCEP·HCl) at 37 °C. The progress of the reaction was monitored by RP-HPLC and the reaction reached completion in 4 h, indicating that the ligation reaction proceeded efficiently even at the sterically demanding junction site of Ile–Cys (Fig. 3b). It is notable that only a trace amount of hydrolyzed product of latent crypto-thioester was observed during the ligation. According to the previous Pd chemistry,^21^ the Acm group was completely removed in a one-pot manner by adding 6 M HCl to adjust the pH to 6.0 followed by addition of 40 equiv. of PdCl_2_ at room temperature in 40 min to afford **Aff4** in 54% isolated yield (Fig. 3b). Interestingly, although Acm-deprotected **Aff4** and **Aff2** co-existed in the NCL buffer; no side-reactions such as double ligation of **Aff2** or self-cyclization of **Aff2** were observed. Furthermore, no MPAA thioester of either **Aff4** or **Aff2** was observed, suggesting that these CPMeOxd–Tle crypto-thioesters were still dormant after Acm deprotection. This phenomenon could be explained by a high affinity between the Pd(ii) complex and the thiol group of Cys, which would function as “transient protecting groups”, thereby suppressing the *N*–*S* acyl shift. The second ligation between segment **Aff4** (final conc 2.0 mM) and **Aff3** (final conc 2.5 mM) was performed in NCL buffer at 37 °C (Fig. 3c). The reaction reached completion in 2 h with 53% yield. After free-radical-based desulfurization,^22^ full-length affibody was obtained in 82% isolated yield (Fig. 3d and e).

**Fig. 4:**
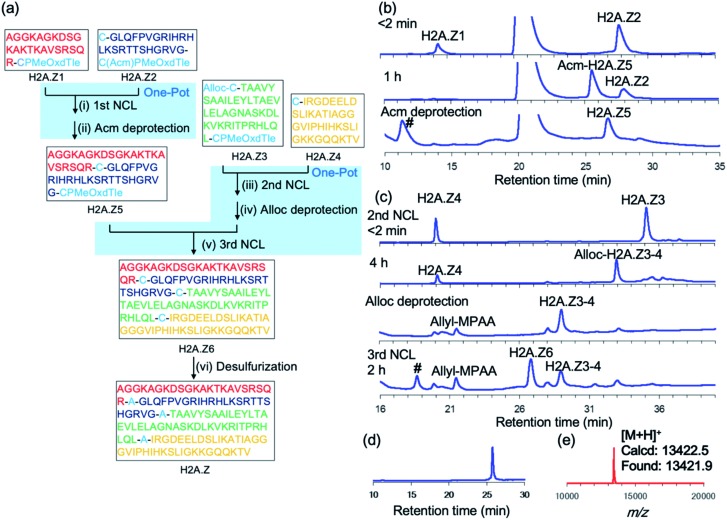
Synthesis of histone H2A.Z by using CPI peptides. (a) Synthetic route. (i) 25 mM TCEP·HCl, 100 mM MPAA, 6 M Gdn·HCl, 0.2 M sodium phosphate (pH 7.0) 37 °C, 1.5 h. (ii) 40 equiv. PdCl_2_, room temperature, 40 min, 49%. (iii) 25 mM TCEP·HCl, 100 mM MPAA, 6 M Gdn·HCl, 0.2 M sodium phosphate (pH 7.0) 37 °C, 4 h. (iv) 3.0 equiv. Pd/TPPTS, room temperature, 1 h. (v) 37 °C, 2 h, 45%. (vi) 500 mM TCEP·HCl, 500 mM glutathione, 150 mM VA-044, 6 M Gdn·HCl, 0.2 M sodium phosphate (pH 7.0), 37 °C, 1 h, 31%. (b) First ligation between **H2A.Z1** and **H2A.Z2**. # = non-peptidic products. (c) The second ligation, Alloc deprotection and third ligation. # = **H2A.Z5–H2A.Z4** ligated product. (d) Analytical HPLC trace and (e) MALDI-MS of purified full-length H2A.Z.

We next synthesized histone H2A.Z, a variant of histone H2A. Histone protein, which is a component of the nucleosome, is an attractive synthetic target because its activity is tightly controlled by a large number of post-translational modifications.^23^,^24^ H2A.Z-containing nucleosomes have decreased stability, which facilitates transcription activation, repair, and chromosomal domain segregation.^25^ We replaced Ala with Cys at positions 23, 48, and 89 to divide the full-length H2A.Z (127 aa) into four segments: **H2A.Z1** (1–22), **H2A.Z2** (23–47), **H2A.Z3** (48–88), and **H2A.Z4** (89–127). To conduct a convergent ligation of these peptide segments as shown in Fig. 4a, the C-terminus of the fragments **H2A.Z1** and **H2A.Z3** was prepared as the reactive crypto-thioester CPMeOxd–Tle and the fragment **H2A.Z2** was synthesized as the latent crypto-thioester C(Acm)PMeOxd–Tle. The N-terminus Cys of fragment **H2A.Z3** was protected with an allyloxycarbonyl (Alloc) group to make the second and third ligations in a one-pot approach according to our previous study24*i* (Fig. 4a). MALDI-MS analysis of the products of the initial synthesis of segments **H2A.Z1** and **H2A.Z3** revealed that by-products with a decreased mass of 18 Da were dominantly obtained, which could be attributed to aspartimide formation from aspartate during MeOxd formation (Fig. S22†). To suppress aspartimide formation, Fmoc–Asp(O^*t*^Bu)–Ser[psi(Me,Me)Pro]^26^ at the Asp8–Ser9 site in segment **H2A.Z1** and Fmoc–Asp(OBno)–OH^27^ at Asp75 in segment **H2A.Z3** were used. As a consequence, aspartimide formation was minimized in the production of the desired segments (Fig. S23a and c†). With the four segments in hand, the first ligation between **H2A.Z1** (final conc 2.0 mM) and **H2A.Z2** (final conc 2.5 mM) was conducted in NCL buffer at 37 °C. The reaction reached completion in 2 h, and subsequent Acm deprotection was performed in one pot with 40 equiv. of PdCl_2_ at room temperature in 40 min, to obtain segment **H2A.Z5** with an isolated yield of 49% (Fig. 4b). The second ligation, Alloc deprotection, and the third ligation were then conducted in a one-pot manner. The second ligation was conducted by ligating **H2A.Z3** (final conc 2.5 mM) and **H2A.Z4** (final conc 2.0 mM) in NCL buffer at 37 °C in 4 h. Following NCL, Alloc deprotection was performed by adding 3 equiv. of Pd/TPPTS complex at room temperature for 1 h (ref. 24*i*) (Fig. 4c). The third ligation was conducted by adding **H2A.Z5** (1–47) and the reaction was allowed to proceed at 37 °C for 2 h, giving 45% isolated yield from **H2A.Z4** (Fig. 4c). Finally, free-radical desulfurization was performed to give the full length of histone H2A.Z in 31% isolated yield (Fig. 4d and e).

**Fig. 5:**
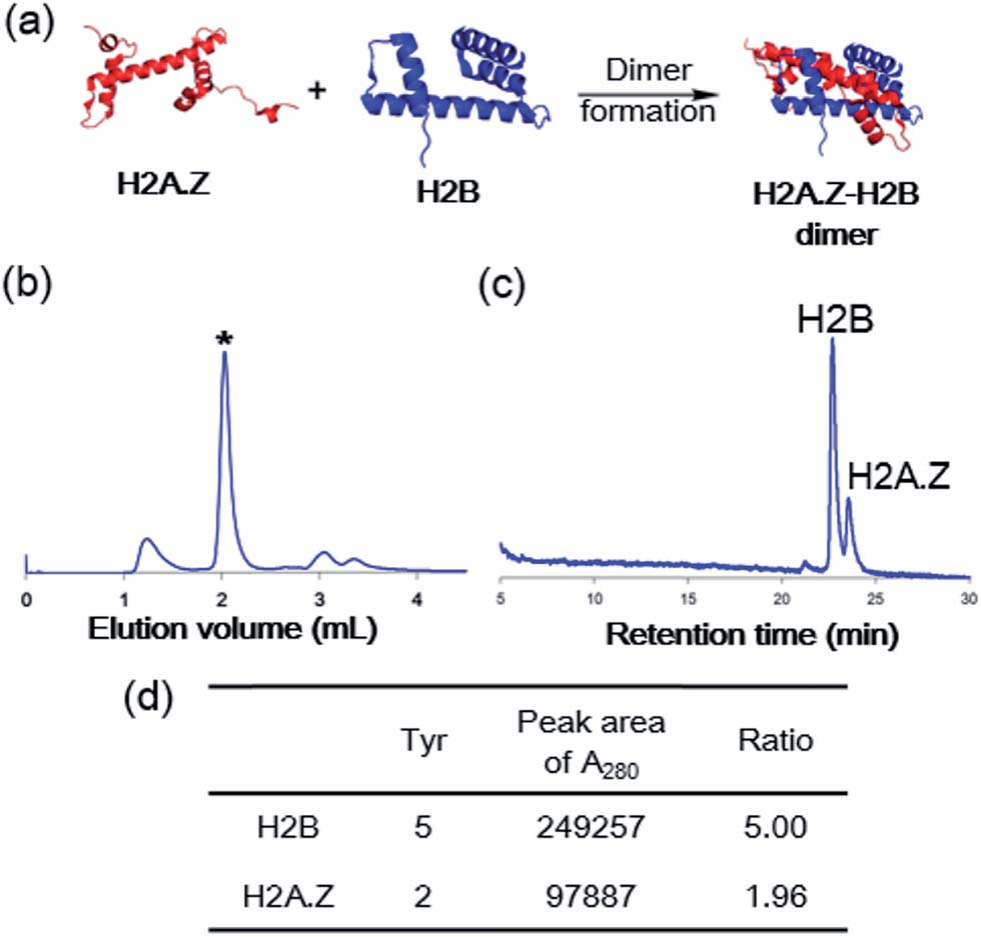
(a) Histone dimer formation of recombinant H2A.Z or synthetic H2A.Z with H2B (Protein Data Bank entry 5B33). (b) Size-exclusion chromatography analysis of the reconstituted mixture. * = H2A.Z–H2B dimer. (c) HPLC trace of the reconstituted synthetic H2A.Z–H2B dimer at 280 nm. (d) Evaluation of the ratio of H2B to H2A.Z based on the number of tyrosine molecules calculated from the HPLC peak area at 280 nm.

To confirm the biophysical properties of the synthetic H2A.Z, we reconstituted the heterodimer of H2A.Z–H2B (Fig. 5). The synthetic H2A.Z and the recombinant H2B were dissolved in an unfolding buffer (7 M Gdn·HCl, 20 mM mercaptoethanol, 20 mM Tris·HCl, pH 7.5). After incubation for 2 h on ice, the solution was dialyzed three times against a refolding buffer (2.0 M NaCl, 2.0 mM mercaptoethanol, 10 mM Tris·HCl, pH 7.5) at 4 °C for 3 h. Size-exclusion chromatography of the reconstituted mixture and subsequent RP-HPLC analysis showed that the heterodimer was obtained in a ratio of 1 : 1 (Fig. 5b–d).

## Conclusions

We have developed a novel C-terminal crypto-thioester, CPMeOxd–Tle, which rapidly transforms into a thioester at neutral pH. This crypto-thioester can be directly synthesized on a solid support and the reactivity is completely suppressed by protecting the mercapto group of Cys. Fast thioesterification rates in the reactive state and high stability in the latent state were achieved by fine-tuning the structure of the leaving group.

The practicality of CPMeOxd–Tle for protein chemistry synthesis was demonstrated by the synthesis of affibody *via* N-to-C ligation, and histone H2A.Z synthesis *via* a convergent synthesis, utilizing the property that the reactivity can be controlled by manipulating the protection/deprotection state of the thiol group of Cys. This crypto-thioester can also be synthesized by using only commercially available reagents, which is a great advantage in protein chemical synthesis.

## Experimental section

### Peptide synthesis of CPNbz/CPMeNbz peptides

For CPNbz peptides, a Dawson Dbz AM resin (47.6 mg, 10 μmol) was used. For CPNbz–Tle, CPMeNbz, and CPMeNbz–Tle peptides, a H-Rink amide TG resin (42 mg, 10 μmol) was used. Dbz/MeDbz units were coupled by using Fmoc–Dbz–OH or Fmoc–MeDbz–OH (4 equiv.) with HBTU (3.9 equiv.)/DIEA (8 equiv.) in DMF (final conc *ca.* 0.1 M). After peptide elongation according to the classical Fmoc-procedure, the resin was washed with DCM, and *p*-nitrochloroformate (10.1 mg, 50 μmol, 5 equiv.) in DCM was added. The resin was vortexed for 40–60 min, and then washed with DCM and DMF, before 5% DIEA in DMF was added. The reaction proceeded for 20 min. After washing the resin with DMF and DCM and final drying, the product was cleaved with a cleavage cocktail (92.5% TFA, 5% TIPS, and 2.5% water) at room temperature for 2 h. The cleavage cocktail solution containing the peptide was added over cold *tert*-butylmethyl ether and precipitated by centrifugation. The supernatant was removed and the residue was dissolved in 0.1% TFA containing water/acetonitrile and purified on a semipreparative column to obtain the desired peptide as a white powder.

### Peptide synthesis of CPOxd/CPMeOxd peptides

For CPOxd peptides and CPMeOxd peptides, Fmoc–Ser(TBDMS)–OH and Fmoc–Thr(TBDMS)–OH were used, respectively. Peptides were elongated on Rink amide resin (42 mg, 10 μmol) by following classical Fmoc procedures. After peptide elongation, the TBDMS group was removed with 1 M TBAF in THF (500 μL). The reaction mixture was stirred for 30 min at room temperature, and then washed with THF, DMF, and water and DMF. For Oxd formation, CDI (16.2 mg, 100 μmol, 10 equiv.) and DIEA (9.6 μL, 100 μmol, 10 equiv.) in DMF (500 μL) were added to the reaction column and the mixture was stirred at room temperature for 8 h. For MeOxd formation, CDI (81.0 mg, 500 μmol, 50 equiv.) and DIEA (48 μL, 500 μmol, 50 equiv.) in DMF (500 μL) were added to the reaction column and the mixture was stirred at room temperature for 24 h. After washing the resin with DMF and DCM and final drying, the product was cleaved with the standard cleavage cocktail.

### Peptide synthesis of CPPyr peptides

For CPPyr peptides, Fmoc–Glu(OAll)–OH was used. After peptide elongation, the allyl group was selectively deprotected with PhSiH_3_ (1 mmol, 123 μL, 20 equiv.) and Pd(PPh_3_)_4_ (20.2 mg, 17.5 μmol, 0.35 equiv.) in CH_2_Cl_2_ (1 mL), and the reaction was allowed to proceed for 30 min at room temperature. The mixture was washed with DCM and DMF. For Pyr formation, CDI (32.4 mg, 200 μmol, 40 equiv.) and DIEA (37.8 μL, 200 μmol, 40 equiv.) in DMF (500 μL) were added to the reaction column and the mixture was stirred at room temperature for 8 h. After washing the resin with DMF and DCM and final drying, the product was cleaved with the standard cleavage cocktail.

### MESNa thioester conversion of CPI peptides or CPE peptides

CPI or CPE peptides (50 nmol, **1**, **2G**, **3**, **4**, **9**, **10**) were dissolved in sodium phosphate buffer (50 μL, 0.1 M, pH 6.0 or 7.3) containing 25 mM tris(2-carboxyethyl)phosphine hydrochloride and 50 mM sodium 2-mercaptoethanesulfonate and incubated at 37 °C. At the indicated time points, aliquots (8.0 μL) of the reaction solution were quenched with 10% TFA/water, and the mixture was analyzed by RP-HPLC. Conversion yield was calculated based on Tyr 280 nm absorbance.

### Native chemical ligation of different CPI peptides

CPI peptides or peptide MESNa thioester (50 nmol, **2A**, **2F**, **2G**, **2K**, **2L**, **2N**, **2Q**, **2S**, **2V**, **2a**) were dissolved in sodium phosphate buffer (50 μL, 0.1 M, pH 7.0) containing 25 mM tris(2-carboxyethyl)phosphine hydrochloride, 100 mM 4-mercaptophenylacetic acid, and 2.0 mM cysteine, and incubated at 37 °C. At the indicated time points, aliquots (8.0 μL) of the reaction solution were quenched with 10% TFA/water, and the mixture was analyzed by RP-HPLC. Conversion yield was calculated based on Tyr 280 nm absorbance.

### Hydrolytic stability assessment of CPI peptides

CPI peptides (50 nmol, **1**, **2G**, **3**, **4**, **6–12**) were dissolved in sodium phosphate buffer (50 μL, 0.1 M, pH 7.0) and incubated at 37 °C. At the indicated time points, aliquots (8.0 μL) of the reaction solution were quenched with 10% TFA/water, and the mixture was analyzed by RP-HPLC. Conversion yield was calculated based on Tyr 280 nm absorbance.

## Conflicts of interest

There are no conflicts to declare.

## Supplementary Material

Supplementary information
