# Supplementary material for: Cysteinylprolyl imide (CPI) peptide: a highly reactive and easily accessible crypto-thioester for chemical protein synthesis
Source: Chem Sci. 2019 May 9;10(23):5967–75. doi: 10.1039/c9sc00646j (PMC6566460; doi:10.1039/c9sc00646j)
Supplement: Supplementary file 1 [file SC-010-C9SC00646J-s001.pdf]

Electronic Supplementary Material (ESI) for Chemical Science.
This journal is © The Royal Society of Chemistry 2019

Supporting Information

Cysteinylprolyl Imide (CPI) Peptide: A Highly Reactive and Easily Accessible Crypto-thioester for Chemical Protein Synthesis

Masafumi Yanase,[a] Koki Nakatsu,[a] Charlane Joy Cardos,[a] Yoshiki Konda,[a] Gosuke Hayashi*[ab] and Akimitsu Okamoto*[ac]

[a]Department of Chemistry and Biotechnology, The University of Tokyo, 7-3-1 Hongo, Bunkyo-ku, Tokyo 113-8656, Japan

[b]Department of Biomolecular Engineering, Graduate School of Engineering, Nagoya University, Furo-cho, Chikusa-ku, Nagoya 464-8603, Japan

[c]Research Center for Advanced Science and Technology, The University of Tokyo, 4-6-1 Komaba, Meguro-ku, Tokyo 153-8904, Japan

Table of Contents

General Information

Experimental Section

1. **Chemical Synthesis of Fmoc-Thr(TBDMS)-OH**
2. **Peptide Synthesis**
3. **Native Chemical Ligation of Different CP*Oxd* Peptides**
4. **Quantitative side-by-side analysis between CPMeOxd-Tle peptide (14) and hydrazide peptide (15)**
5. **Chemical Synthesis of Affibody**
6. **Chemical Synthesis of Histone H2A.Z**

Figure S1. Screening of activator reagents for Cys-Pro-Oxazolidinone formation.

Figure S2. Validation of additive effects for Cys-Pro-Oxazolidinone formation.

Figure S3. Screening of activator reagents for Cys-Pro-Pyrrolidinone formation.

Table S1. Sequence and calculated and observed mass of the used model peptides.

Figure S4. Direct ethyldisulifide protection form trtyl-protected cysteine.

Figure S5. HPLC trace of purified peptides.

Figure S6. HPLC assessment of stereoisomer of peptide **2G**.

Figure S7. MESNa thioesterification of CPNbz peptide **1**.

Figure S8. MESNa thioesterification of CPOxd peptide **2G**.

Figure S9. MESNa thioesterification of CPPyr peptide **3**.

Figure S10. MESNa thioesterification of CPE peptide **4**.

Figure S11. NCL of CPOxd peptides with cysteine.

Figure S12. NCL of CPOxd peptide **2a** with cysteine.

Figure S13. Stability assessment of CPNbz peptide **1**.

Figure S14. Stability assessment of CPOxd peptide **2G**.

Figure S15. Stability assessment of CPOxd/CPMeOxd peptides (**610**).

Figure S16. Stability assessment of peptides CPMeNbz (**11**) and CPNbz-Tle (**12**).

Figure S17. MESNa thioesterification of CPMeOxd peptides **9** and **10**.

Figure S18. NCL kinetics analysis of peptides CPMeOxdTle (**13**) and MESNa thioester (**5A**).

Figure S19. NCL kinetics analysis of peptides CPMeOxdTle (**14**).

Figure S20. NCL kinetics analysis of peptide hydrazide (**15**).

Figure S21. HPLC trace and MALDI-MS of pure Affibody fragment.

Figure S22. MALDI-MS of crude of initial synthesis of fragment **H2A.Z1** and **H2A.Z3**.

Figure S23. HPLC trace and MALDI-MS of pure H2A.Z fragment.

General Information

All Fmoc-protected amino acids were purchased from Watanabe chemical, Novabiochem, Peptide institute, inc. and other commercial buyers. Other chemicals were purchased from Sigma-Aldrich, Wako chemicals, Tokyo Chemical Industry, and other commercial buyers. All solvents and reagents were commercially available and used without further purification. MALDI-MS measurements were performed by using microflex (BRUKER) system.

Preparative HPLC purifications of peptides were carried out by using PU-2080 plus Intelligent HPLC Pump (JASCO) and MD-2018 plus Photodiode Array Detector (JASCO) at 195 to 650 nm and AR-II column (20 ID x 250) for short peptides or Protein-R (20 ID x 250) for long peptides from Nacalai was used with a binary mixture of A (0.1% TFA, 99.9% H2O) and B (0.1% TFA, 99.9% acetonitrile) as a mobile phase (flow rate = 6.0 mL/min) in a linear gradient as described. For analytical HPLC measurements of peptides, AR-II column (4.6 ID x 250) or Protein-R column (4.6 ID x 250) from Nacalai was used with a binary mixture of A (0.1% TFA, 99.9% H2O) and B (0.1% TFA, 99.9% acetonitrile) as a mobile phase (flow rate = 1.0 mL/min) in a linear gradient as described.

Experimental Section

1. **Chemical Synthesis of Fmoc-Thr(TBDMS)-OH.**

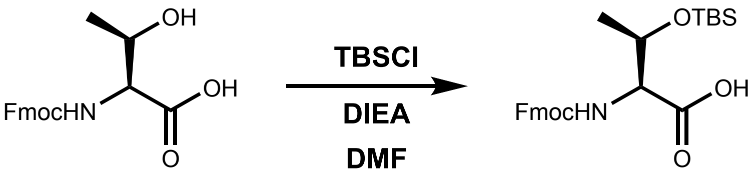

The product was prepared based on the previous literature.[1] Briefly, Fmoc-Thr-OH (1.71 g, 5 mmol, 1 equiv) was dissolved in 10 mL DMF and DIPEA (2.8 mL, 16 mmol, 3.2 equiv). TBSCl (1.5 g, 10 mmol, 2 equiv) was then added. The reaction was stirred at r.t. for 24 h. The mixture was acidified to pH 3~4 with 1 M HCl, extracted with EtOAc and washed with brine twice. The organic phase was dried over NaSO4 ,concentrated and purified by column chromatography (Hexane: EtOAc = 6:1 to 3:1 with 1% AcOH) to give the product as a white solid (1.22 g, 54%). 1H-NMR (600 MHz, Chloroform-D) δ 7.80 (d, J = 7.8 Hz, 2H), 7.64 (q, J = 3.5 Hz, 2H), 7.44 (t, J = 7.6 Hz, 2H), 7.35 (t, J = 7.3 Hz, 2H), 5.64 (d, J = 7.3 Hz, 1H), 4.42-4.51 (m, 3H), 4.36 (q, J = 3.5 Hz, 1H), 4.28 (t, J = 7.1 Hz, 1H), 1.23 (d, J = 6.4 Hz, 3H), 0.95 (s, 9H), 0.18 (s, 6H).

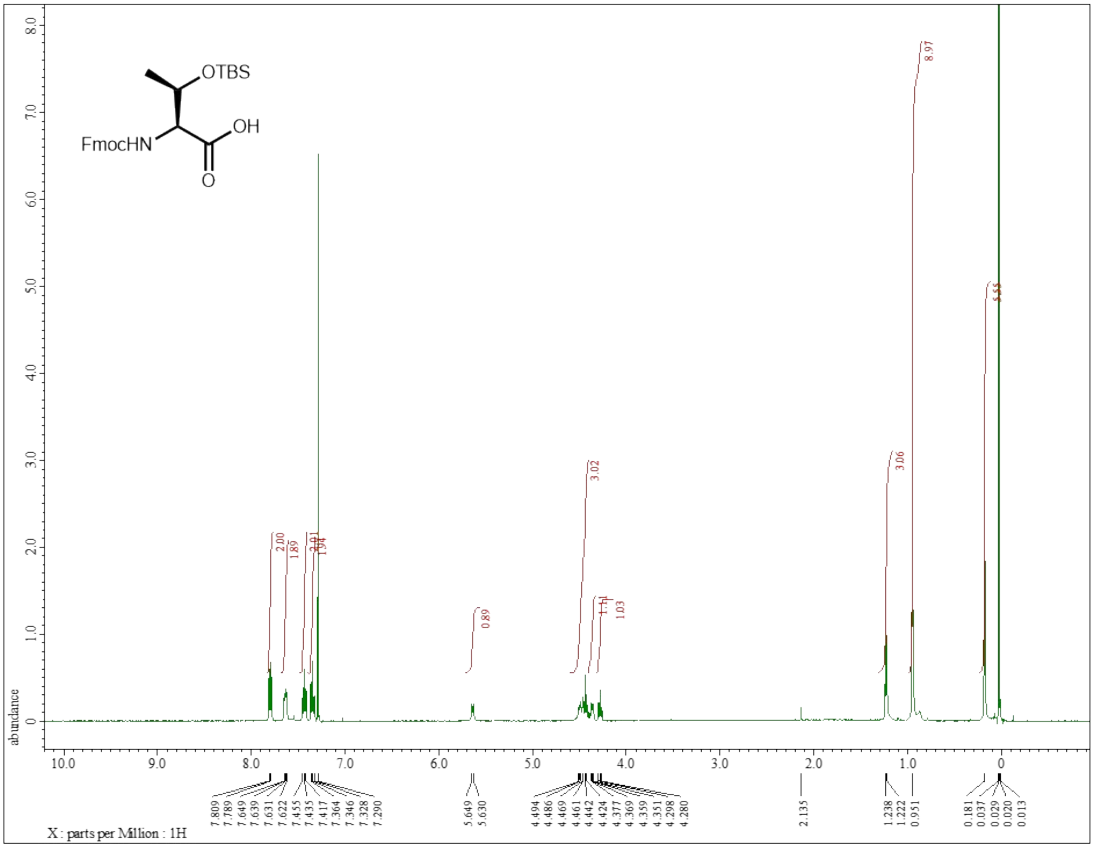
OTBS
OH
FmocHN
O
abundance
X : parts per Million : 1H

2. Peptide Synthesis

Solid phase peptide synthesis (SPPS)

Fmoc Amino Acid Alko-PEG resin (Watenabe chemical) was used for CO_2H-terminal peptides. TentaGel Resin (0.25 mmol/g, HiPep Lab.) was used for C-terminal NH_2 peptides.

Automated solid-phase peptide synthesis was performed by using a MultiPep RS (Intavis) using the usual Fmoc-SPPS protocol. Amino acids protected by 9-fluorenylmethoxycarbonyl (Fmoc) group were coupled with *O*-(1*H*-Benzotriazol-1-yl)-*N*,*N*,*N′*,*N′*-tetramethyluronium hexafluorophosphate (HBTU) as activator and N,N-diisopropylethylamine (DIEA) as base.

Manual solid-phase peptide synthesis was performed by the following protocol:

Coupling of amino acids other than His and Cys derivatives: Fmoc-protected amino acids (4 equiv) were activated with HBTU (3.9 equiv) and DIEA (8 equiv) in DMF (final conc ca. 0.1 M) and transferred to the resin (coupling time: 30 min at 40 °C or 60 min at room temperature).

Coupling of His and Cys derivatives: Fmoc-protected amino acids (4 equi) were activated with DIC (4 equiv) and OxymaPure (4 equiv) in NMP (final conc ca. 0.1 M) and transferred to the resin (coupling time: 60 min at room temperature).

For Fmoc deprotection, the resin was treated with 20% piperidine in DMF for 5 minutes twice.

Cleavage cocktail: 92.5% TFA, 5% TIPS, 2.5% water was added. After 2 h, the solution was filtered. The crude peptides were obtained by precipitation with cold ether and centrifugation. The crude peptide was dissolved in a mixture of 0.1%TFA-containing water and 0.1%TFA-containing acetonitrile, purified by preparative HPLC and analyzed by MADLI-MS.

Cys-Pro-Ester (CPE) peptides.

CPE peptide was prepared based on the previous literature.[2] The peptide was assembled on a H-Rink amide TG resin (42 mg, 10 µmol) by Fmoc-SPPS according to classical procedure. For glycolic acid coupling, glycolic acid (3.0 mg, 40 µmol, 4 equiv), N,N'-diisopropylcarbodiimide (DIC) (6.2 µL, 40 µmol, 4 equiv), and 1-hydroxybenzotriazole (HOBt) (6.5 mg, 40 µmol, 4 equiv) in NMP (0.4 ml) was added to the resin. The resin was vortexed overnight at room temperature. For Gly-Cys coupling, Fmoc-Gly-Cys(Trt)-OH (25.6 mg, 40 µmol, 4 equiv), diisopropylcarbodiimide (DIC) (6.2 µL, 40 µmol, 4 equiv), and 3-hydroxy-1,2,3-benzotriazin-4-one (HOOBt) (6.5 mg, 40 µmol, 4 equiv) in NMP (0.4 ml) was added to the resin. The resin was vortexed for 2 h at 40 °C. The resin was treated with 10%Ac2O and 5% DIEA in NMP for 5 min. After peptide elongation, the resin was washed with DMF and DCM. The peptide was cleaved from the resin by the 500 µL of the reagent cocktail (87.5% TFA, 5% TIPS, 2.5% water and 5% diethyl disulfide) at room temperature for 2h. For the protection of mercapto group of Cys during peptide cleavage, the cleavage cocktail contains diethyldisulfide. The cleavage cocktail solution containing the peptide was added over cold *tert*-buthylmethyl ether and precipitated by centrifugation. The supernatant was removed and the residue was dissolved in 0.1%TFA containing water/acetonitrile and purified on a semipreparative column to obtain the desired peptide as a white powder.

3. Native Chemical Ligation of Different CP*Oxd* and Thioester Peptides

CPI peptides or peptide MESNa thioester (50 nmol, **2A**, **2F**, **2G**, **2K**, **2L**, **2N**, **2Q**, **2S**, **2V**, **2a**) were dissolved in the sodium phosphate buffer (50 µL, 0.1 M, pH 7.0) containing 25 mM tris(2-carboxyethyl)phosphine hydrochloride, 100 mM 4-mercaptophenylacetic acid and 2.0 mM

cysteine and incubated at 37°C. At the indicated time points, 8.0 μL aliquots of the reaction solution was quenched by 10% TFA-containing water, and analyzed by RP-HPLC. Conversion yield was calculated based on Tyr 280 nm absorbance.

Determination of the apparent second order kinetic constants.

Kinetics study condition was as follows: 1.0 mM peptide (**5A** or **13**), 2.0 mM cysteine, 25 mM MPAA, 50 mM TCEP･HCl, 6 M Gdn･HCl, 0.2 M sodium phosphate (pH 7.1), 37°C.

Kinetic analysis was conducted considering the following equation:

$$\underset{A}{\text{Peptide-CPMeOxdTle or Peptide-MESNa thioester}} + \underset{B}{\text{Cys}} \xrightarrow{k_{app}} \underset{C}{\text{Peptide-Cys}}$$

Following the general rate equation for $[A_0] \neq [B_0]$:

$$kt = \frac{1}{[B_0] - [A_0]} \ln\left(\frac{[A_0][B]}{[B_0][A]}\right)$$

4. Quantitative side-by-side analysis between CPMeOxd-Tle peptide (13) and hydrazide peptide (14).

Solid phase peptide synthesis. CPMeOxd-Tle peptide (**14**) was synthesized as mentioned earlier. Hydrazide peptide (**15**) was synthesized according to the previous reports.[3] Briefly, Cl-Trt(2-Cl) resin was swelled in 50% DCM/DMF for 20 min. After draining of the solvent, 5% hydrazine in DMF (1.0 mL) was added to the resin and the mixture was agitated for 1 h at rt. The solvent was drained and washed with DMF. This operation was repeated again. Then, the resin

was washed with DMF, DCM and DMF. Next, 5% MeOH/DMF (1.0 mL) was added and stirred for 10 min. Then, the resin was washed with DMF, DCM and DMF. Immediately, Fmoc-Gly (4 equiv.), HATU (3.9 equiv.) and DIEA (8.0 equiv.) in DMF were added to the resin. The mixture was stirred for 1 h at rt. Resin loading was determined by the absorbance of the filtrate at 301 nm (ε = 7800 L mol^{-1} cm^{-1}) during Fmoc-cleavage of Fmoc-Gly-attached resin. After peptide elongation, the peptide was cleaved from the resin by the cleavage cocktail (1.0 mL, TFA/TIPS/water 92.5/5.0/2.5) for 2 h at rt. Then, the resin was filtered and the filtrate was precipitated by pre-cooled methyl *t*butyl ether (10 mL). Ether was decanted and the crude precipitation was dried, dissolved in 0.1%TFA containing water/acetonitrile and purified by RP-HPLC.

Yields: CPMeOxd-Tle peptide (**14**) 6.74 mg (4.84 μmol, 43%), Hydrazide peptide (**15**) 8.10 mg (8.38 μmol, 63%).

NCL with cysteine.

NCL of CPMeOxd-Tle peptide (**14**) with cysteine was conducted as follows: CPMeOxd-Tle peptide (**14**, 2.09 mg, 1.50 μmol) was dissolved in 1.5 mL of NCL buffer (25 mM TCEP･HCl, 100 mM MPAA, 0.1 M sodium phosphate, pH 7.0) containing cysteine (2 mM). The reaction mixture was vortexed and incubated at 37°C. At each time points, 8.0 μL of reaction solution was quenched by 10 μL of 10% TFA/water, diluted with 80 μL of water, analyzed and purified by RP-HPLC. Isolated yield: 1.20 mg (1.14 μmol, 76%).

NCL of hydrazide peptide (**15**) with cysteine was conducted as follows: Hydrazide peptide (**15**, 1.52 mg, 1.57 μmol) was dissolved in 0.75 mL of 0.2 M phosphate buffer (pH 3.0) containing 6 M guanidine hydrochloride and the solution was cooled at -15 °C. Then, 75 μL of 0.5 M $NaNO_2$ was added to the solution and the reaction solution was incubated at -15 °C for 15 min. Then, to

the reaction solution, 0.75 mL of NCL buffer (200 mM MPAA, 0.2 M sodium phosphate, pH 7.0) containing cysteine (4 mM) was added and pH was adjusted to 7.0 with 6 M NaOH and vortexed. The reaction solution was incubated at 37 °C. At each time points, 8.5 μL of reaction solution was quenched by 10 μL of 10% TFA/water. To monitor the ligation reaction, 80 μL of 0.1 M TCEP in phosphate buffer (0.1 M, pH 7.0) was added and incubated at rt for 10 min. After the peptide ligation has proceeded for 2 h, 500 μL of 0.1 M TCEP･HCl in phosphate buffer (0.1 M, pH 7.0) was added and incubated for 10 min and purified by RP-HPLC. Isolated yield: 0.93 mg (0.88 μmol, 58%).

5. Chemical Synthesis of Affibody

Fragment synthesis. [Ala1-Ile16]-CPMeOxdTle-NH_2 peptide (**Aff1**) and [Cys17-Arg28]-C(Acm)PMeOxdTle-NH_2 peptide (**Aff2**) were assembled on a Fmoc-NH-SAL-PEG resin. [Cys29-Lys58]-OH peptide (**Aff3**) was assembled on a Fmoc-Lys(Boc)-Alko-PEG Resin. For Aff1 synthesis, Fmoc-C(Trt)-OH as Cys in CPI was used. Boc-amino acids were used at Ala1 and Cys17. Peptides were elongated by peptide synthesizer (MultiPep RS (Intavis)). After peptide ligation, TBDMS group was deprotected by 1 M TBAF in THF for 30 minutes at room tempature. Then, it was washed with THF, DMF, water and DMF. For MeOxd formation, 1, 1'-carbonyldiimidazole (50 equiv), DIEA (50 equiv) in DMF was added to the reaction column and stirred at room temperature for 24 h. The resin was washed with DMF and CH2Cl2. The peptide was cleaved from the resin the reagent cocktail (1 mL): 92.5% TFA, 5% TIPS and 2.5% water at room temperature for 2h. The cleavage cocktail solution containing the peptide was added over cold tert-buthylmethyl ether and precipitated by centrifugation. The supernatant was removed and the residue was dissolved in 0.1%TFA containing water/acetonitrile and purified on a

semipreparative column to obtain the desired peptide as a white powder. Yields were calculated based on the loading of first Fmoc-amino acid (Fmoc-Tle-OH) or capacity of the Fmoc-Lys(Boc)-Alko-PEG Resin. Yields: **Aff1** 2.26 mg (0.787 μmol, 7.9%), **Aff2** 4.95 mg (2.12 μmol, 17%), and **Aff3** 10.9 mg (2.65 μnol, 13%).

1st NCL and Acm removal (one-pot). [Ala1-Ile16]-CPMeOxdTle-NH_2 peptide (**Aff1**) (1.45 mg, 0.53 μmol) and [Cys17-Arg28]-C(Acm)PMeOxdTle-NH_2 peptide (**Aff2**) (3.3 mg, 0.75 μmol) was dissolved in 0.265 mL of NCL buffer (25 mM TCEP･HCl, 100 mM MPAA, 6M Gdn･HCl, 0.2 M sodium phosphate (pH 7.0)). The reaction was carried out at 37 °C. After the ligation completed (4 h), 5.8 μL of 6 M HCl aq. was added to the reaction solution to adjust pH ~6. Then, Na_2PdCl_4･$3H_2O$ (9.2 mg, 40 equiv) was added to the solution. The reaction mixture was stirred for 40 min at room temperature (about 22°C). The product (**Aff4**) was isolated by HPLC and characterized by MALDI-MS. Before analysis and isolation of Acm deprotection reaction, 20 times the volume of 1,2-dithiothreitol solution (1M) was added to the reaction solution to release the peptide from the Palladium species. The isolated yield was 54% (1.28 mg, 0.28 μmol).

2nd NCL. [Ala1-Arg28]-CPMeOxdTle-NH_2 peptide (**Aff4**) (0.53 mg, 0.12 μmol) and [Cys29-Lys58]-OH peptide (**Aff3**) (0.63 mg, 0.15 μmol) was dissolved in 0.059 mL of NCL buffer (25 mM TCEP･HCl, 100 mM MPAA, 6M Gdn･HCl, 0.2 M sodium phosphate (pH 7.0)). The reaction was carried out at 37 °C. After the ligation completed (2.5 h), the product (**Aff5**) was isolated by HPLC and characterized by MALDI-MS. The isolated yield was 53% (0.50 mg, 0.062 μmol).

Desulfurization. [Ala1-Lys58]-OH peptide (**Aff5**) (0.50 mg, 0.062 μmol) was dissolved in 0.062 mL of desulfurization buffer (500 mM TCEP･HCl, 500 mM glutathion, 150 mM VA-044, 6M Gdn･HCl, 0.2 M sodium phosphate (pH 7.0)). The reaction was carried out at 37 °C. After the

ligation completed (1 h), the full-length of affibody was isolated by HPLC and characterized by MALDI-MS. The isolated yield was 82% (0.40 mg, 0.051 μmol).

6. Chemical Synthesis of Histone H2A.Z

Fragment Synthesis. [Ala1-Arg22]-CPMeOxdTle-NH2 peptide (**H2A.Z1**) and [Cys23-Gly47]-C(Acm)PMeOxdTle-NH2 peptide (**H2A.Z2**), Alloc-[Cys48-Leu88]-CPMeOxdTle-NH2 peptide (**H2A.Z3**) were assembled on a Fmoc-NH-SAL-PEG resin. [Cys89-Lys127]-OH peptide (**H2A.Z4**) was assembled on a Fmoc-Val-Alko-PEG Resin. For **H2A.Z1** and **H2A.Z3** synthesis, Fmoc-Cys(Trt)-OH as Cys in CPI was used. Boc-amino acids were used at Ala1 and Cys23 and Alloc-Cys(Trt)-OH was used at Cys48. Fmoc-Asp(OtBu)-Ser[psi(Me,Me)Pro] at the Asp8-Ser9 and Fmoc-Asp(OBno)-OH at Asp75 was used to prevent aspartimide formation. Peptides were elongated by peptide synthesizer (MultiPep RS (Intavis)). After peptide ligation, TBDMS group was deprotected by 1 M TBAF in THF for 30 minutes at room tempature. Then, it was washed with THF, DMF, water and DMF. For MeOxd formation, 1,1'-carbonyldiimidazole (50 equiv), DIEA (50 equiv) in DMF was added to the reaction column and stirred at room temperature for 24 h. The resin was washed with DMF and DCM. The peptide was cleaved from the resin the reagent cocktail (1 mL): 92.5% TFA, 5% TIPS and 2.5% water at room temperature for 2h. The cleavage cocktail solution containing the peptide was added over cold *tert*-buthylmethyl ether and precipitated by centrifugation. The supernatant was removed and the residue was dissolved in 0.1%TFA containing water/acetonitrile and purified on a semipreparative column to obtain the desired peptide as a white powder. Yields were calculated based on the loading of first Fmoc-amino acid (Fmoc-Tle-OH) or capacity of the Fmoc-Val-Alko-PEG Resin. Yields: **H2A.Z1** 6.22 mg (1.88 μmol, 12%), **H2A.Z2** 9.48 mg (2.18 μmol, 18%), **H2A.Z3** 5.35 mg (0.94 μmol, 6.0%)

and **H2A.Z4** 8.72 mg (1.69 μmol, 8.5%).

1st NCL and Acm removal (one-pot). [Ala1-Arg22]-CPMeOxdTle-NH2 peptide (**H2A.Z1**, 2.0 mg, 0.60 μmol) and [Cys23-Gly47]-C(Acm)PMeOxdTle-NH2 peptide (**H2A.Z2**, 3.3 mg, 0.75 μmol) was dissolved in 0.30 mL of NCL buffer (25 mM TCEP·HCl, 100 mM MPAA, 6 M Gdn·HCl, 0.2 M sodium phosphate (pH 7.0)). The reaction was carried out at 37 °C. After the ligation completed (1.5 h), 4.0 μL of 6 M HCl aq. was added to the reaction solution to adjust pH ~6. Then, Na2PdCl4·3H2O (10.4 mg, 40 equiv) was added to the solution. The reaction mixture was stirred for 40 min at room temperature (about 22°C). The product (**H2A.Z5**) was isolated by HPLC and characterized by MALDI-MS. Before analysis and isolation of Acm deprotection reaction, 20 times the volume of 1,2-dithiothreitol solution (1M) was added to the reaction solution to release the peptide from the Palladium species. The isolated yield was 49% (2.06 mg, 0.29 μmol).

Preparation of Pd/TPPTS complex. Pd/TPPTS complex was prepared just before use according to the previous literature.[4] N,N-dimethylformamide and neat water were purged by argon beforehand. Palladium(II) acetate was dissolved in N,N-dimethylformamide (200 mM) and voltexed for 5-10 min to dissolve completely. 3,3′,3″-Phosphanetriyltris(benzenesulfonic acid) trisodium salt (TPPTS) was dissolved in neat water (1.0 M). 10 μL of 200 mM $Pd(OAc)_2$, 8.0 μL of 1.0 M TPPTS and 2.0 μL of neat water were mixed and vigorously vortexed for 30 s at room temperature under argon atmosphere. The solution can be stored at least for one day at –15 °C under argon atmosphere in the dark.

2nd NCL, Alloc removal and 3rd NCL (one-pot). Alloc-[Cys48-Leu88]-CPMeOxdTle-NH2 (**H2A.Z3**) (1.0 mg, 176 nmol) and [Cys89-Lys127]-OH (**H2A.Z4**) (0.79 mg, 153 nmol) was dissolved in 75 μL of NCL buffer (25 mM TCEP·HCl, 100 mM MPAA, 6M Gdn·HCl, 0.2 M

sodium phosphate (pH 7.0)). The reaction was carried out at 37 °C. After the ligation completed (4 h), 4.0 μL of 6 M HCl aq. was added to the reaction solution to adjust pH ~6. Then, 6.4 μL of Pd/TPPTS (100 mM) was added to the solution. After 1 h, [Ala1-Gly47]-CPMeOxdTle-NH2 (**H2A.Z5**) (0.97 mg, 138 nmol) was added to the solution to allow to react for 2 h at 37°C. Before analysis and isolation of Alloc deprotection reaction, 10 times the volume of MESNa (0.5M) in water was added to the reaction solution to release the peptide from the Palladium species. The isolated yield was 45% (1.16 mg, 69.4 nmol).

Desulfurization. H2A.Z6 (0.80 mg, 47.9 nmol) was dissolved in 47.9 μL of desulfurization buffer (500 mM TCEP・HCl, 500 mM glutathione, 150 mM VA-044, 6M Gdn・HCl, 0.2 M sodium phosphate (pH 7.0)). The reaction was carried out at 37 °C. After the ligation completed (2 h), the full-length of H2A.Z was isolated by HPLC and characterized by MALDI-MS. The isolated yield was 31% (0.25 mg, 15.0 nmol).

Reconstitution of H2A.Z-H2B dimer. Equimolar synthetic histone H2A.Z and recombinant histone H2B were dissolved to a concentration of approximately 1.0 mg/mL in unfolding buffer (20 mM Tris・HCl (pH 7.5), 7 M Gdn·HCl, and 20 mM 2-mercaptoethanol). After the denaturation by incubation for 3 h on ice, the mixture was dialyzed against 1 L of a dialysis buffer (10 mM Tris-HCl (pH 7.5), 2 M NaCl, and 2 mM 2-mercaptoethanol) at 4 °C three times with an Oscillatory Cup molecular weight cutoff 8000 instrument (Cosmo Bio, BTE-212949). The mixture was concentrated with an Amicon Ultra instrument (3 K, 0.5 mL) and then purified by size-exclusion chromatography with a Superdex 200 Increase 5/150 GL column. The histone stoichiometry was confirmed by HPLC analysis.

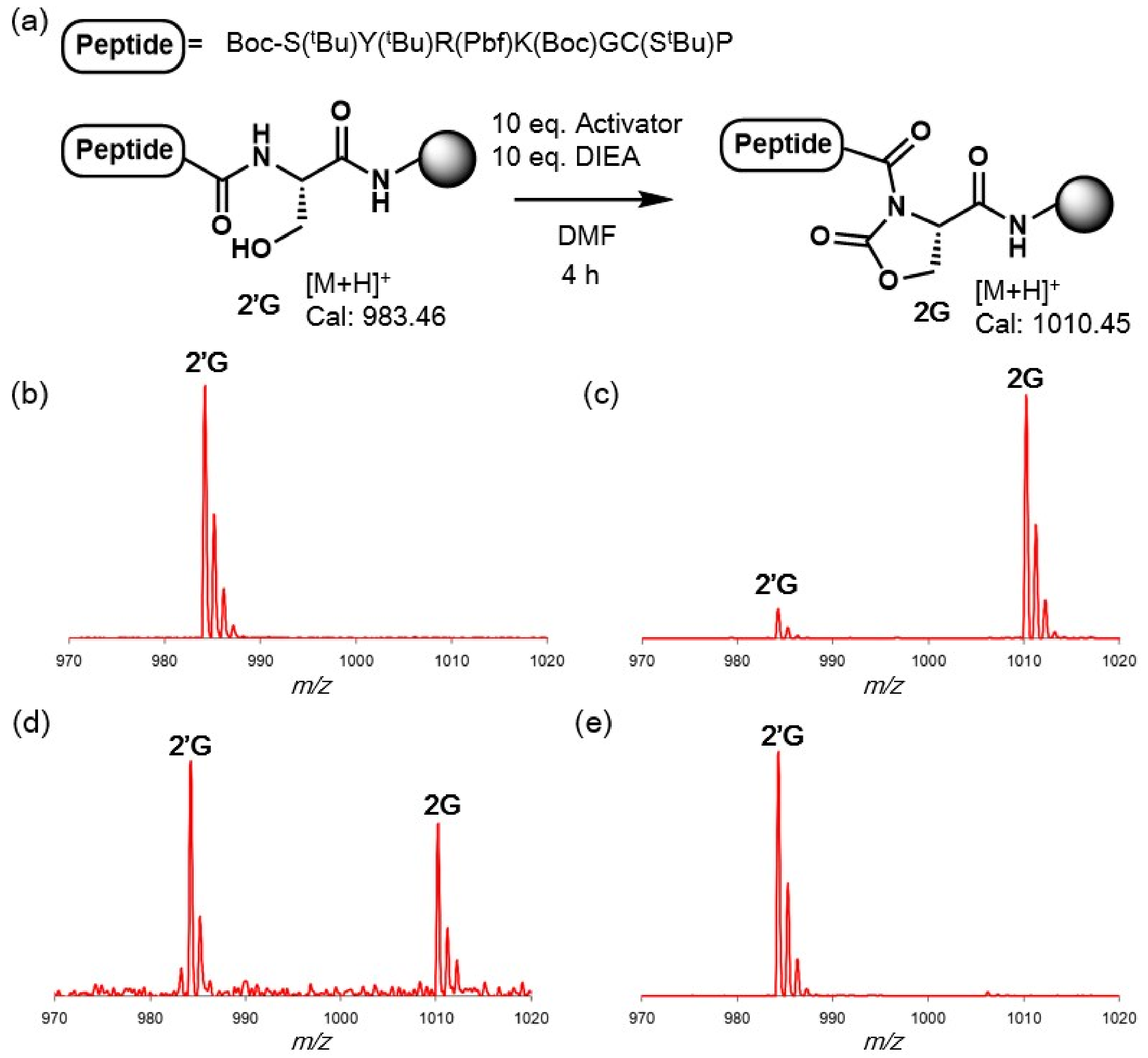

Figure S1. Screening of activator reagents for Cys-Pro-Oxadolidinone formation. (a) Reaction scheme of this reaction. (b)-(e) MALDI-MS of crude peptides before and after activation using different activators. (b) Before activation. (c) 1,1'-Carbonyldiimidazole (CDI). (d) *N*,*N*-Disuccinimidyl Carbonate (DSC), and (e) 4-Nitrophenyl chloroformate (NPC).

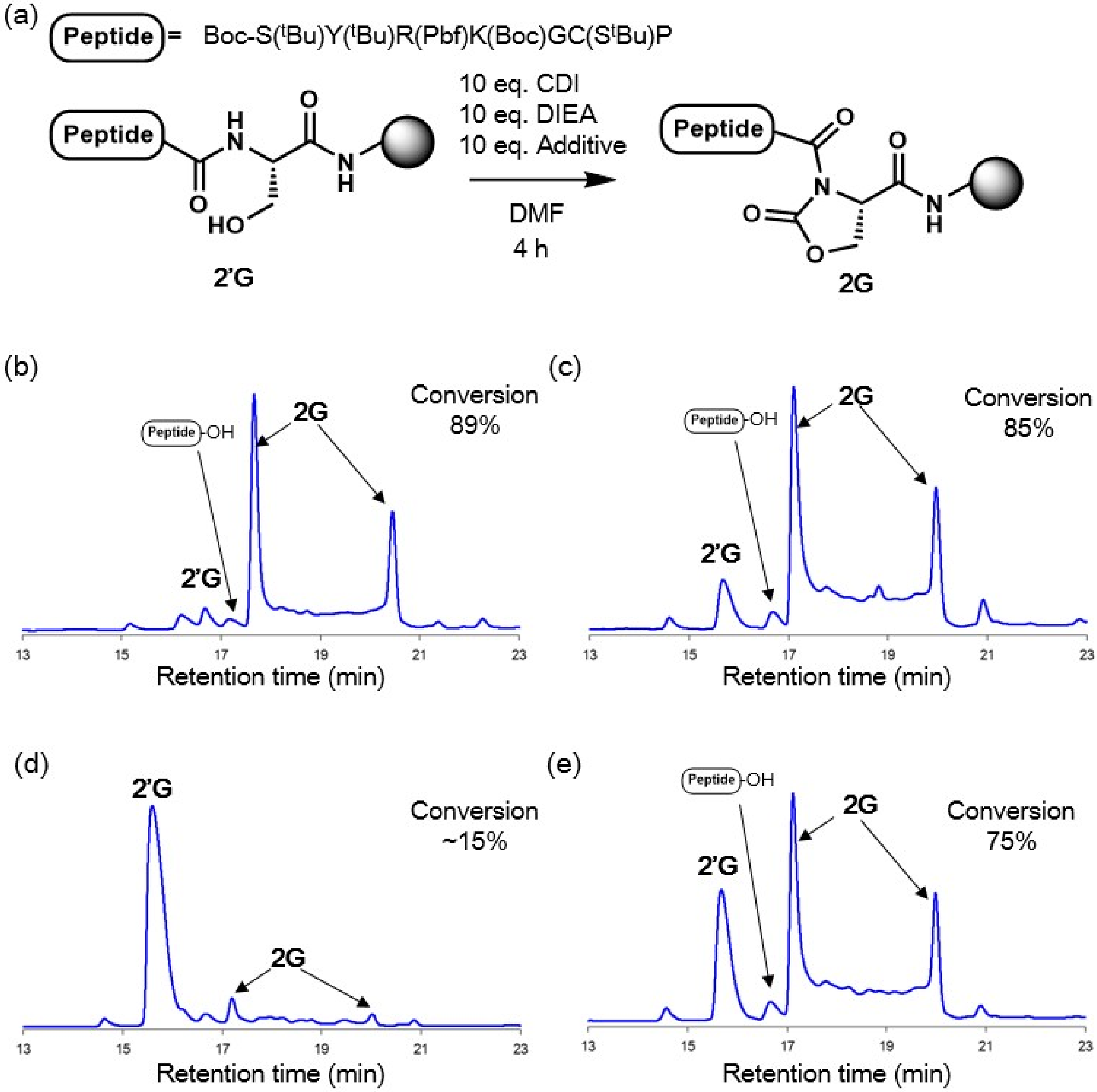

Figure S2. Validation of additive effects for Cys-Pro-Oxadolidinone formation. (a) Reaction scheme of this reaction. (b)-(e) HPLC charts of crude peptides with or without different additives. (b) Without additive. (c) *N*,*N*-Dimethyl-4-aminopyridine. (d) *N*-Hydroxysuccinimide. (e) Pyridine.

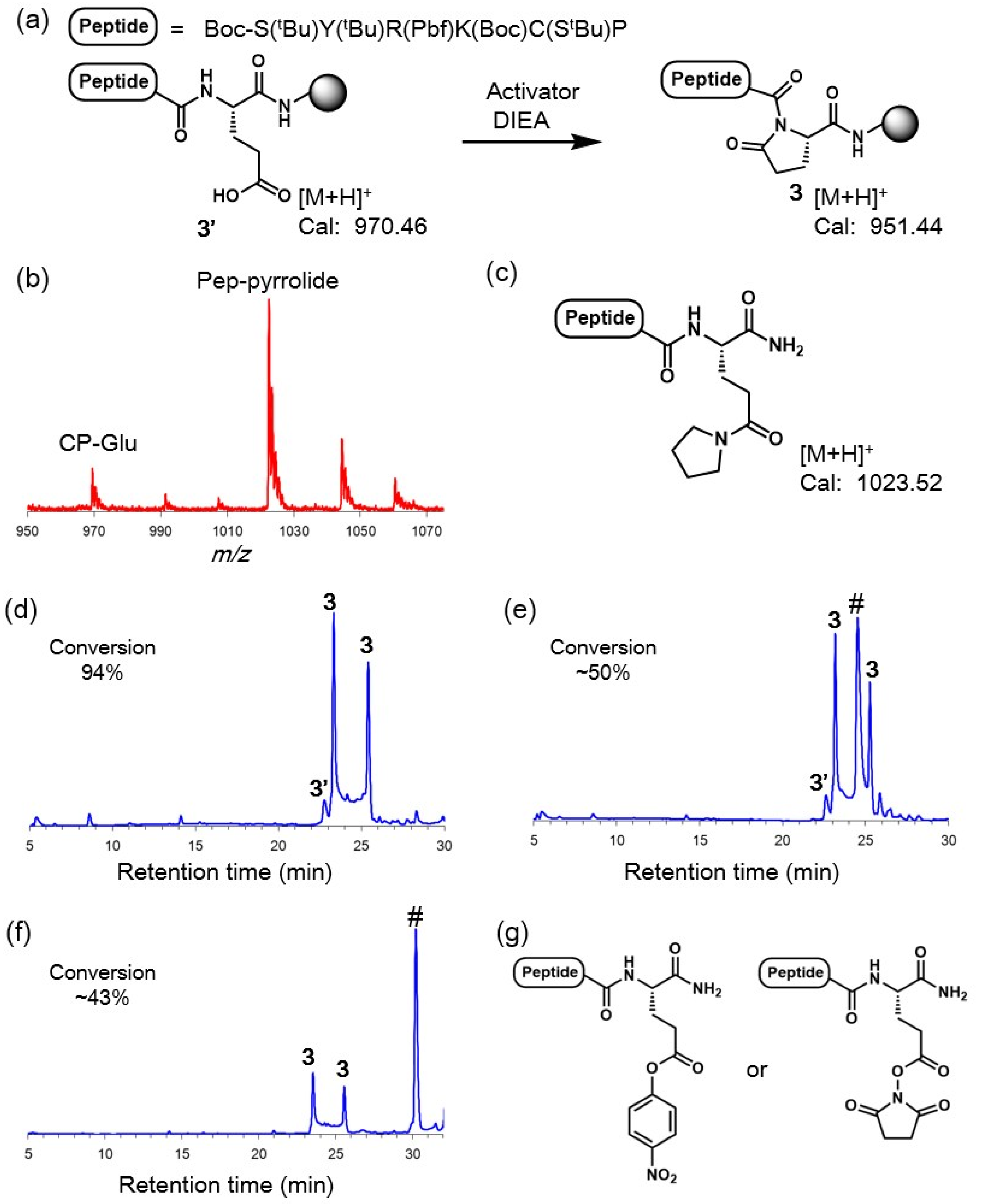

Figure S3. Screening of activator reagents for Cys-Pro-Oxadolidinone formation. (a) Reaction

scheme of this reaction. (b) MALDI-MS of crude peptides following the protocol in the previous study[5]: 10 equiv PyBrop (without any purification and crystalization), 20 equiv DIEA and 2 equiv DMAP in NMP, for 14 h. (c) Molecular structure of the corresponding pyrrolide. (d)-(g) Screening of other activator reagents for Cys-Pro-Oxadolidinone formation. Reaction condition: 40 equiv of activator, 40 equiv DIEA in DMF, for 24 h. HPLC charts of crude peptides activated by (d) 1,1'-Carbonyldiimidazole (CDI), (e) *N*,*N*-Disuccinimidyl Carbonate or (f) 4-Nitrophenyl carbonate. # = Activated ester (g) Molecular structure of the activated esters.

Table S1. Sequence and calculated and observed mass of the used model peptides.

Name	Sequence	[M+H]+	
		Calcd.	Found.
**1**	SYRKGC(StBu)PNbz-NH_2	1057.47	1057.56
**2A**	SYRKAC(StBu)POxd-NH_2	1024.46	1024.94
**2F**	SYRKFC(StBu)POxd-NH_2	1100.49	1100.50
**2G**	SYRKGC(StBu)POxd-NH_2	1010.45	1010.48
**2L**	SYRKLC(StBu)POxd-NH_2	1081.52	1081.90
**2N**	SYRKNC(StBu)POxd-NH_2	1066.51	1066.81
**2Q**	SYRKQC(StBu)POxd-NH_2	1067.47	1067.33
**2S**	SYRKSC(StBu)POxd-NH_2	1081.48	1082.17
**2V**	SYRKVC(StBu)POxd-NH_2	1040.46	1041.51
**2a**	SYRKaC(StBu)POxd-NH_2	1052.49	1052.76
**3**	SYRKGC(StBu)PPyr-NH_2	1024.46	1024.52
**4**	SYRKGC(SEt)PEstTle-NH_2	1008.48	1008.46
**5A**	SYRKA-$SCH_2CH_2SO_3H$	1040.50	1040.20
**6**	SYRKGC(StBu)POxdG-NH_2	748.30	748.28
**7**	SYRKGC(StBu)POxdF-NH_2	1067.47	1067.35
**8**	SYRKGC(StBu)POxdTle-NH_2	1157.52	1157.68
**9**	SYRKGC(StBu)PMeOxd-NH_2	1123.53	1123.56
**10**	SYRKGC(StBu)PMeOxdTle-NH_2	1024.46	1024.70
**11**	SYRKGC(StBu)PNbzTle-NH_2	1137.55	1137.90
**12**	SYRKGC(StBu)PMeNbz-NH_2	1170.55	1171.02
**13**	SYRKACPMeOxdTle-NH_2	1071.49	1071.55
**14**	SYRKGCPMeOxdTle-NH_2	1049.52	1049.53
**15**	SYRKG-$NHNH_2$	624.36	624.39

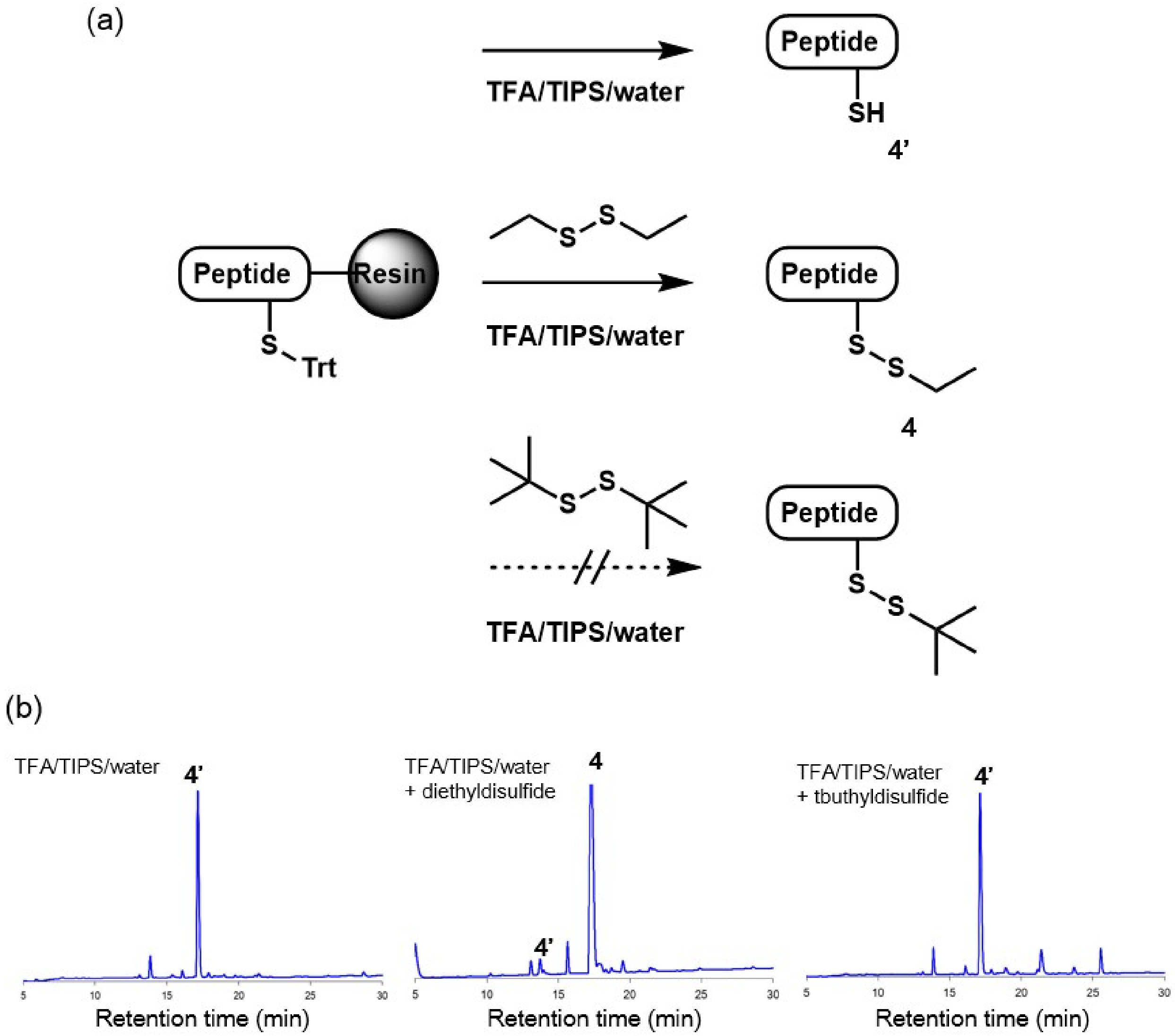

Figure S4. Direct ethyldisulifide protection from trityl-protected cysteine. (a) Reaction scheme. (b) HPLC trace of crude peptides.

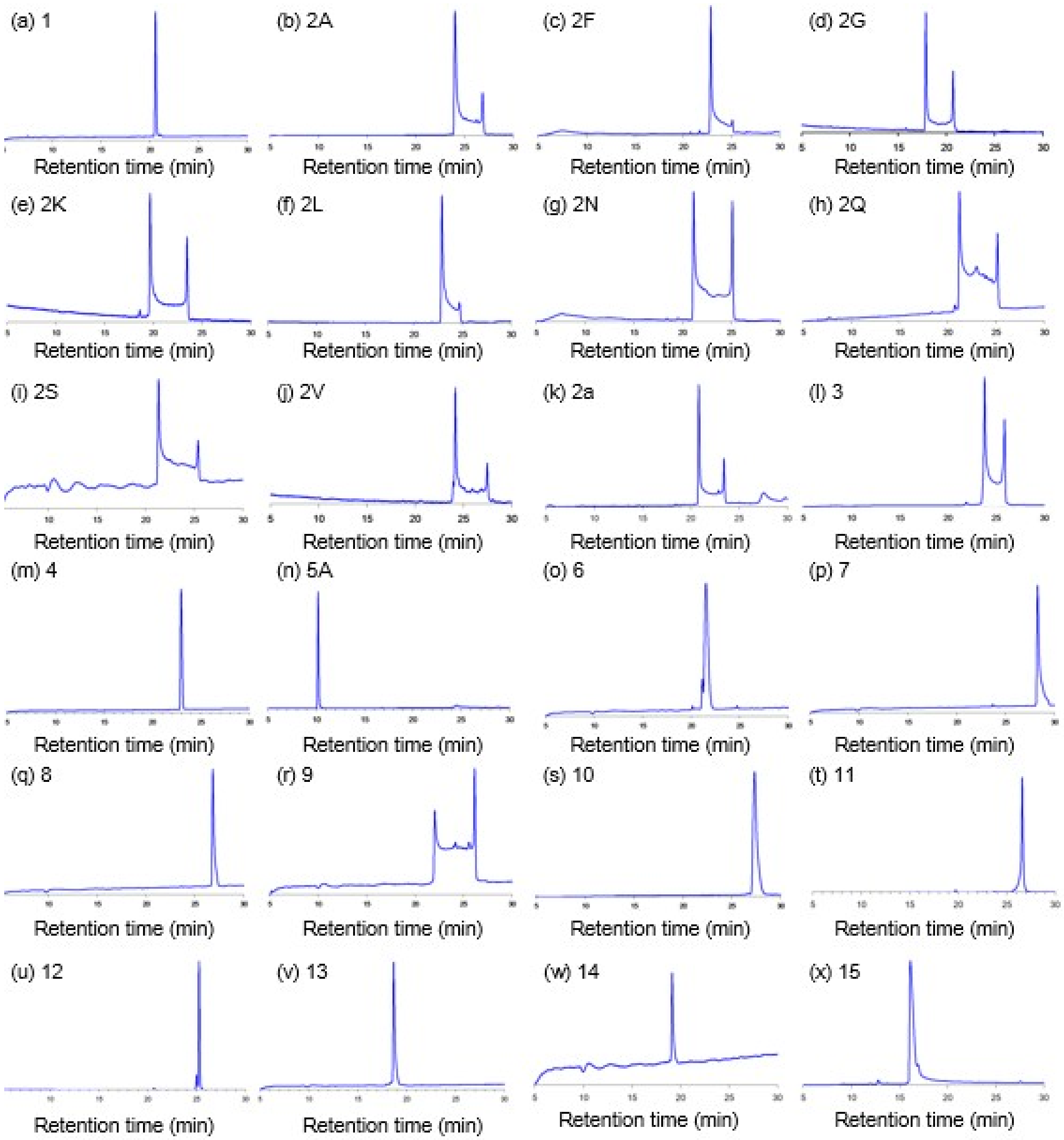

Figure S5. HPLC trace of purified peptides. Peptides were eluted from a AR-II column (4.6×250 mm). Gradient: 5% solution B (acetonitrile/0.1% TFA) in solution A (water/0.1% TFA) to 45% B in A over 30 min (**1**, **2A**, **2F**, **2G**, **2K**, **2L**, **2N**, **2Q**, **2S**, **2a**, **3**, **6**, **7**), 5% to 35% over 30 min (**2V**, **4**, **5A**, **8**, **9**, **10**, **13**, **14**), 5% to 15% to 45% over 10 and 20 min (**11**, **12**) or 1% to 10% over 30 min (**15**). Broad peak which mainly consists of two peaks in the peptides **2A-2V**, **2a, 3** and **9** were assigned to be a mixture of *trans*-*cis* isomers.

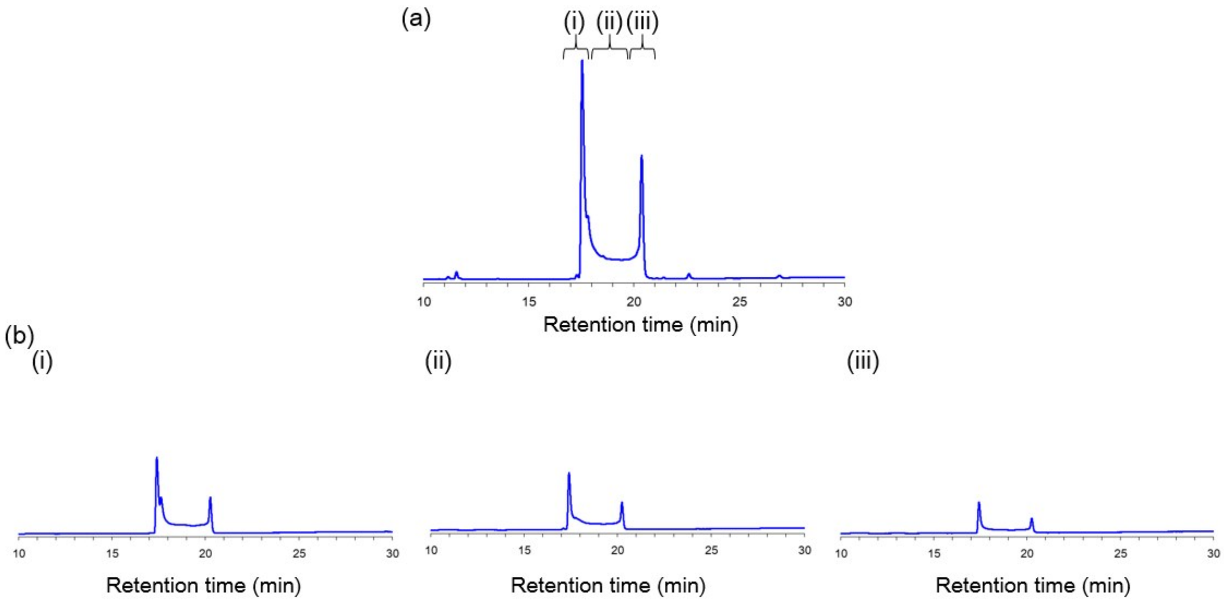

Figure S6. HPLC assessment of stereoisomer of peptide **2G**. (a) HPLC trace of purified peptides (**2G**). (b) Re-injection of three parts of broad peaks of (i) 17.5–17.9 min, (ii) 17.9–20.3 min and (iii) 20.3–20.6 min. Peptides were eluted from a AR-II column (4.6 × 250 mm). Gradient: 5% solution B (0.1% TFA/acetonitrile) in solution A (0.1% TFA/water) to 45% B in A over 30 min.

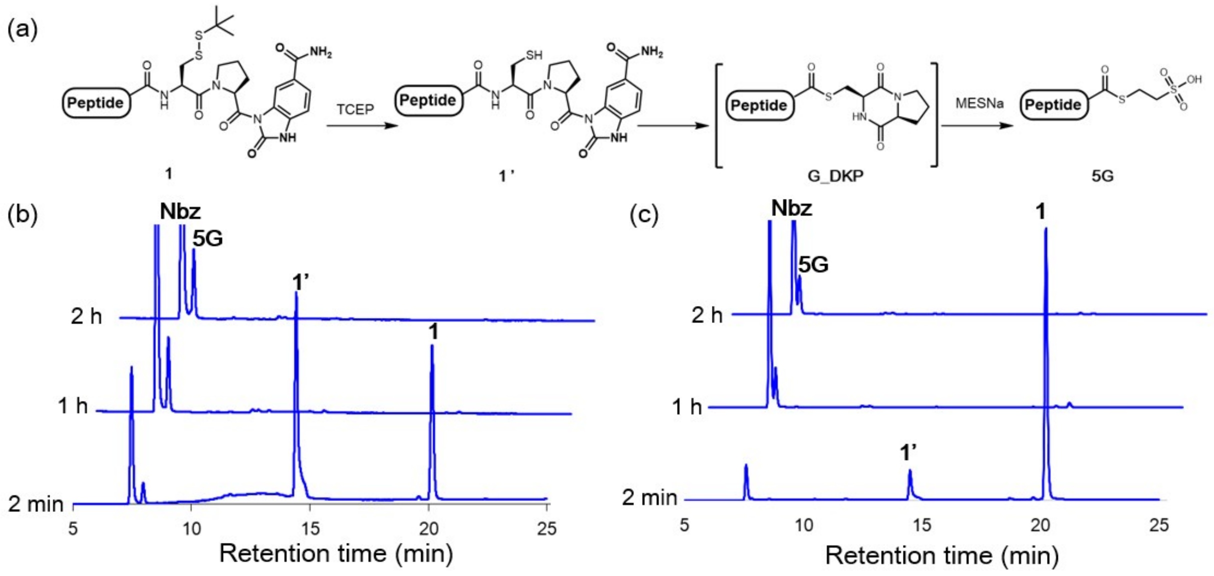

Figure S7. MESNa thioesterification of CPNbz peptide **1**. (a) Reaction scheme of this reaction. HPLC trace of MESNa thioesterification of peptide **1** at each time point at (b) pH 7.3 and (c) pH 6.0.

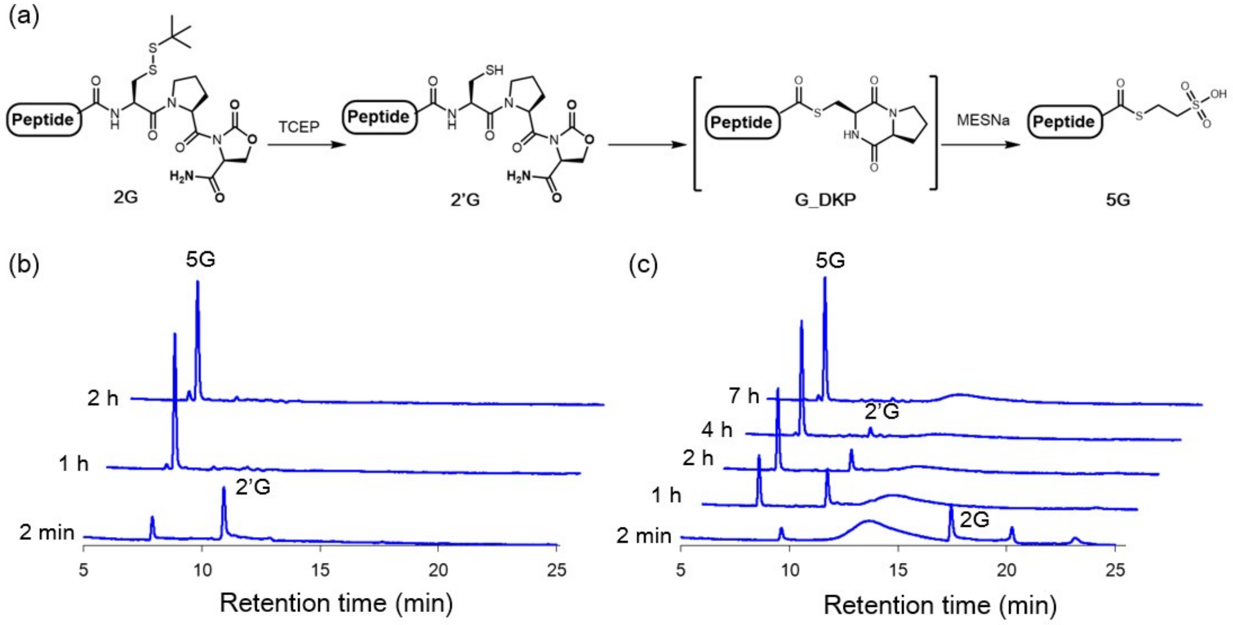

Figure S8. MESNa thioesterification of CPOxd peptide **2G**. (a) Reaction scheme of this reaction. HPLC trace of MESNa thioesterification of peptide **2G** at each time point at (b) pH 7.3 and (c) pH 6.0.

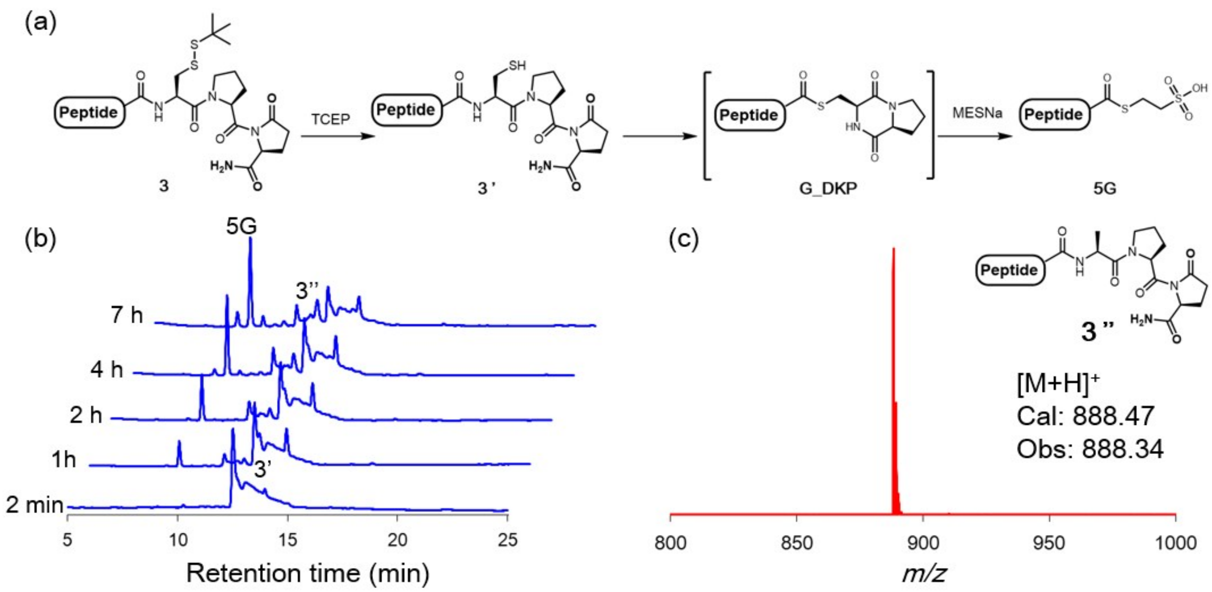

Figure S9. MESNa thioesterification of CPPyr peptide **3**. (a) Reaction scheme of this reaction. (b) HPLC trace of MESNa thioesterification of peptide **3** at each time point at pH 7.3. (c) Molecular structure and MALDI-MS of desulfurized peptide APPyr **3''**.

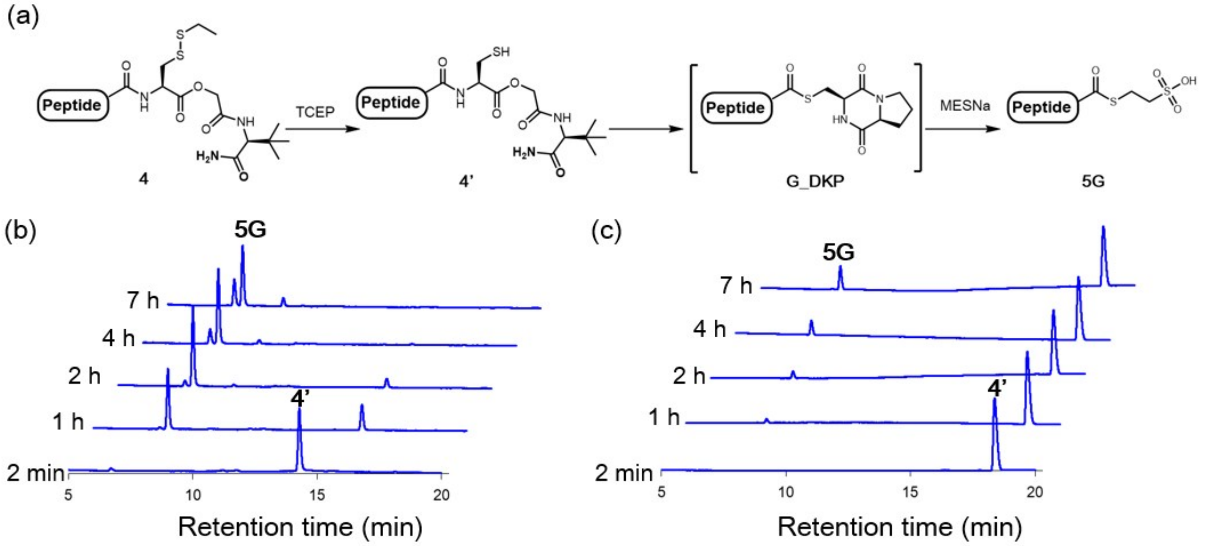

Figure S10. MESNa thioesterification of CPE peptide **4**. (a) Reaction scheme of this reaction. HPLC trace of MESNa thioesterification of peptide **4** at each time point at (b) pH 7.3 and (c) pH 6.0.

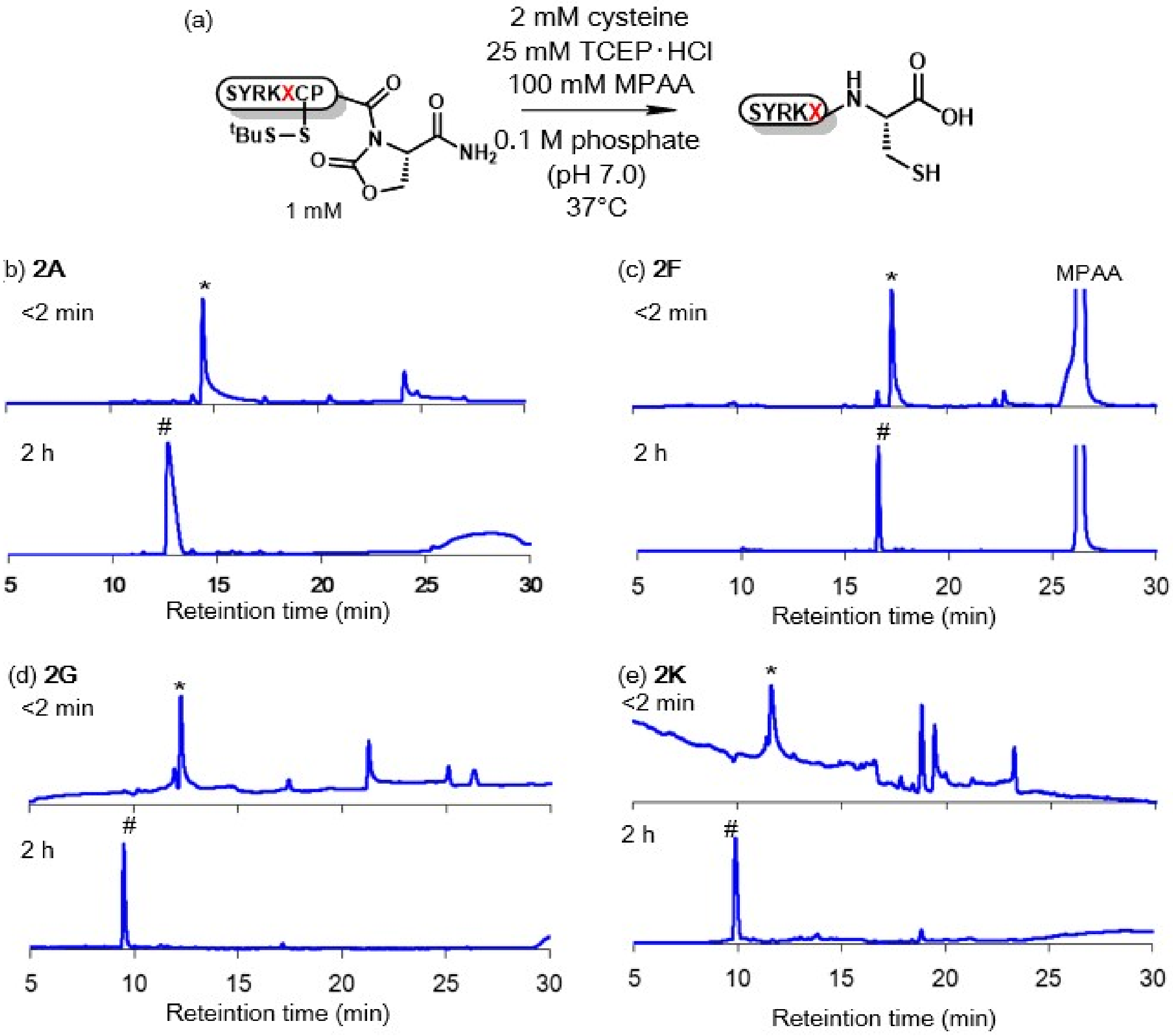
(a)
SYRKXCP
tBuS−S
1 mM
2 mM cysteine
25 mM TCEP·HCl
100 mM MPAA
0.1 M phosphate
(pH 7.0)
37°C
SYRKX
SH
OH
b) 2A
<2 min
2 h
Reteintion time (min)
(c) 2F
MPAA
(d) 2G
(e) 2K

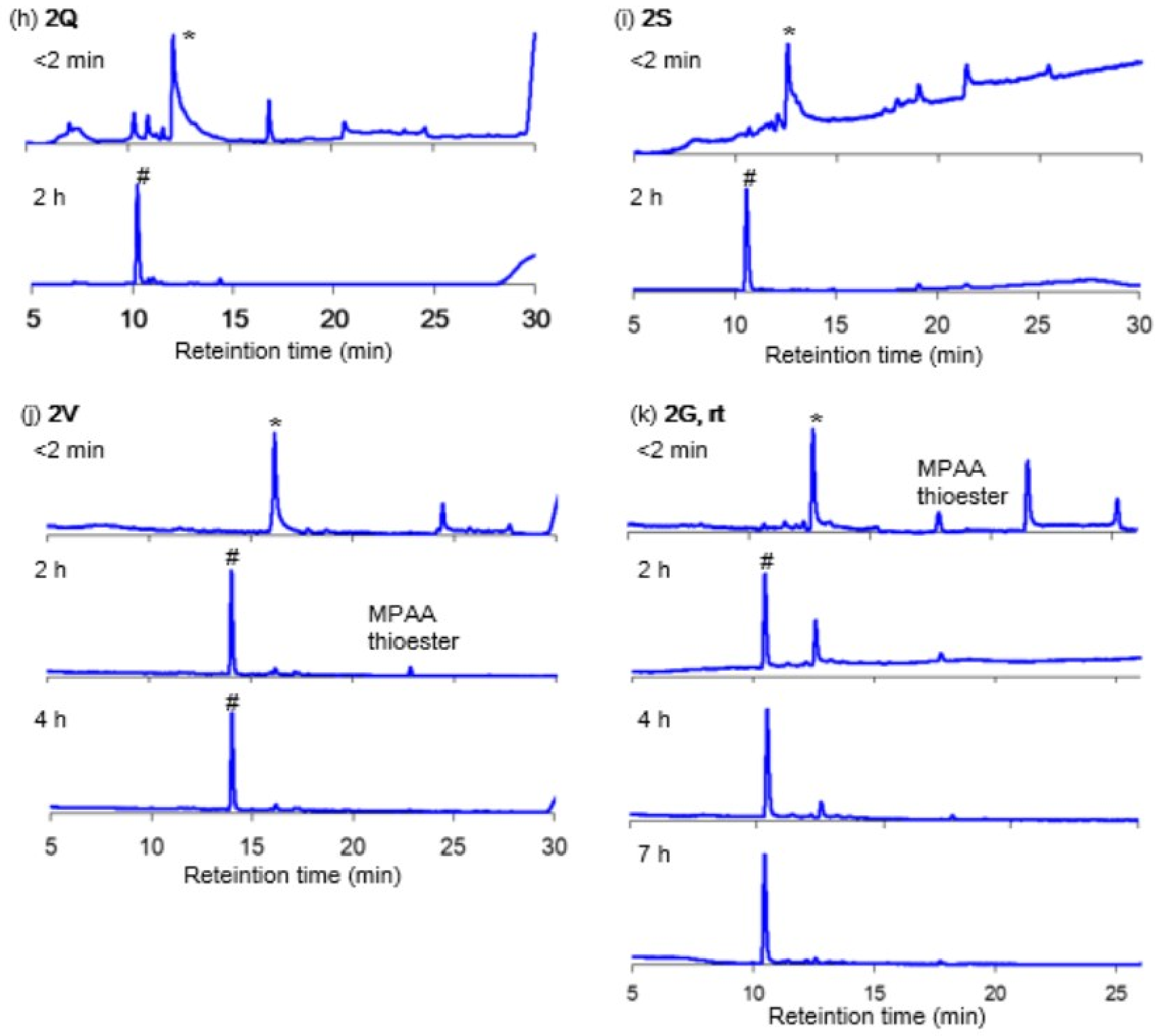

Figure S11. NCL of CPOxd peptides with cysteine. (a) Reaction scheme of this reaction. (b)-(j) HPLC trace of NCL at 37 °C of peptide **2A-2V** at each time point. (k) HPLC trace of NCL at room temperature of peptide **2G** at each time point. * = tButhio-disulfide reduced product. # = ligated product.

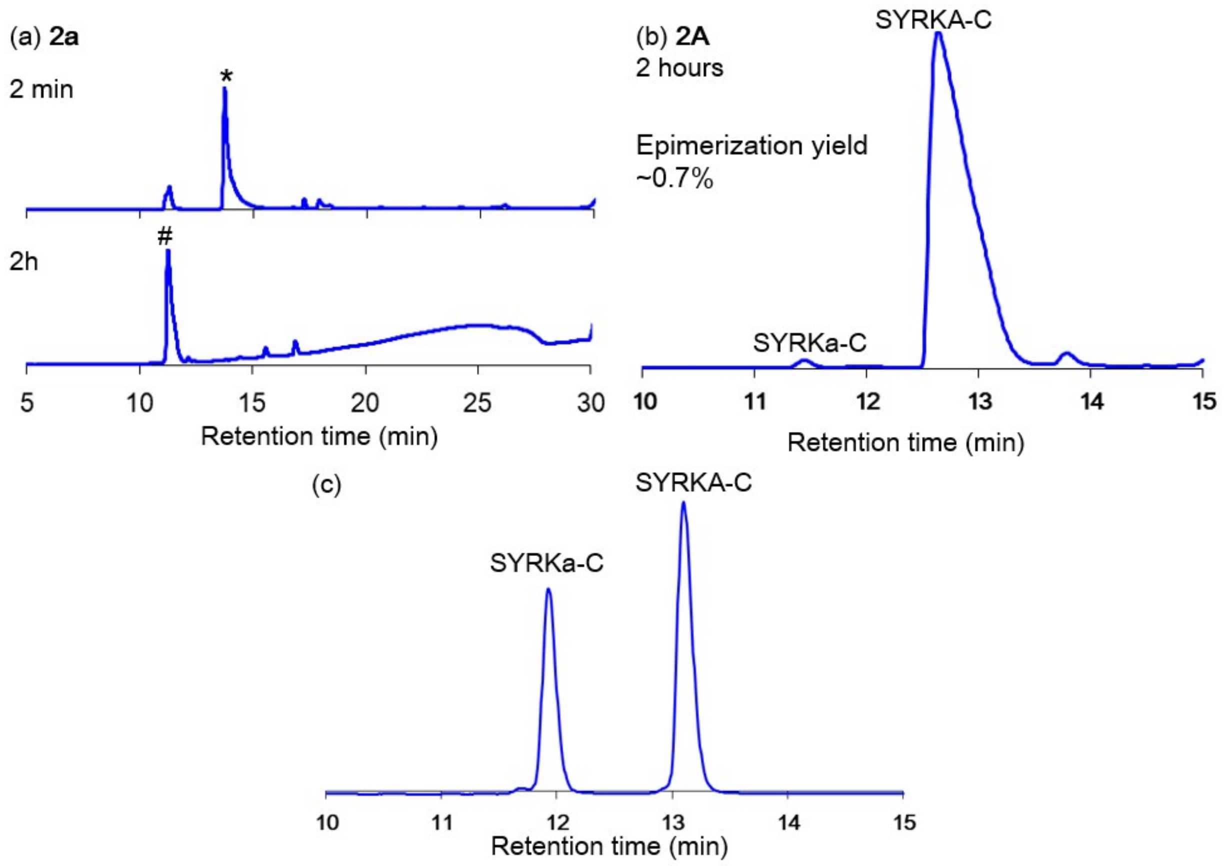

Figure S12. Epimerization analysis of A-CPOxd peptides **2A** with cysteine. (a) HPLC trace of NCL of peptide **2a** at each time point. (b) Expanded HPLC chart of NCL of peptide **2A** at 2h. (c) HPLC trace of co-injection of the ligated product from the peptides **2A** and **2a**.

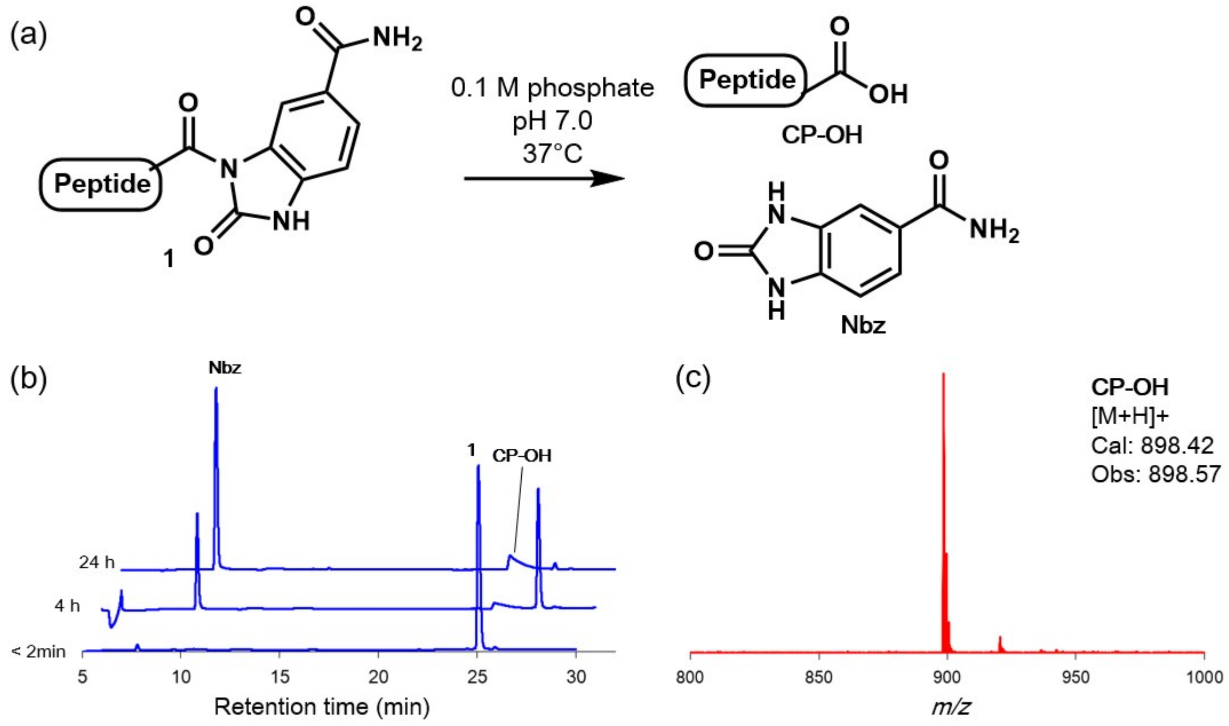

Figure S13. Stability assessment of CPNbz peptide **1**. (a) Reaction scheme of this analysis. (b) HPLC trace of stability assessment of CPNbz peptide at each time point. (c) MALDI-MS of hydrolyzed product peptide **CP-OH**.

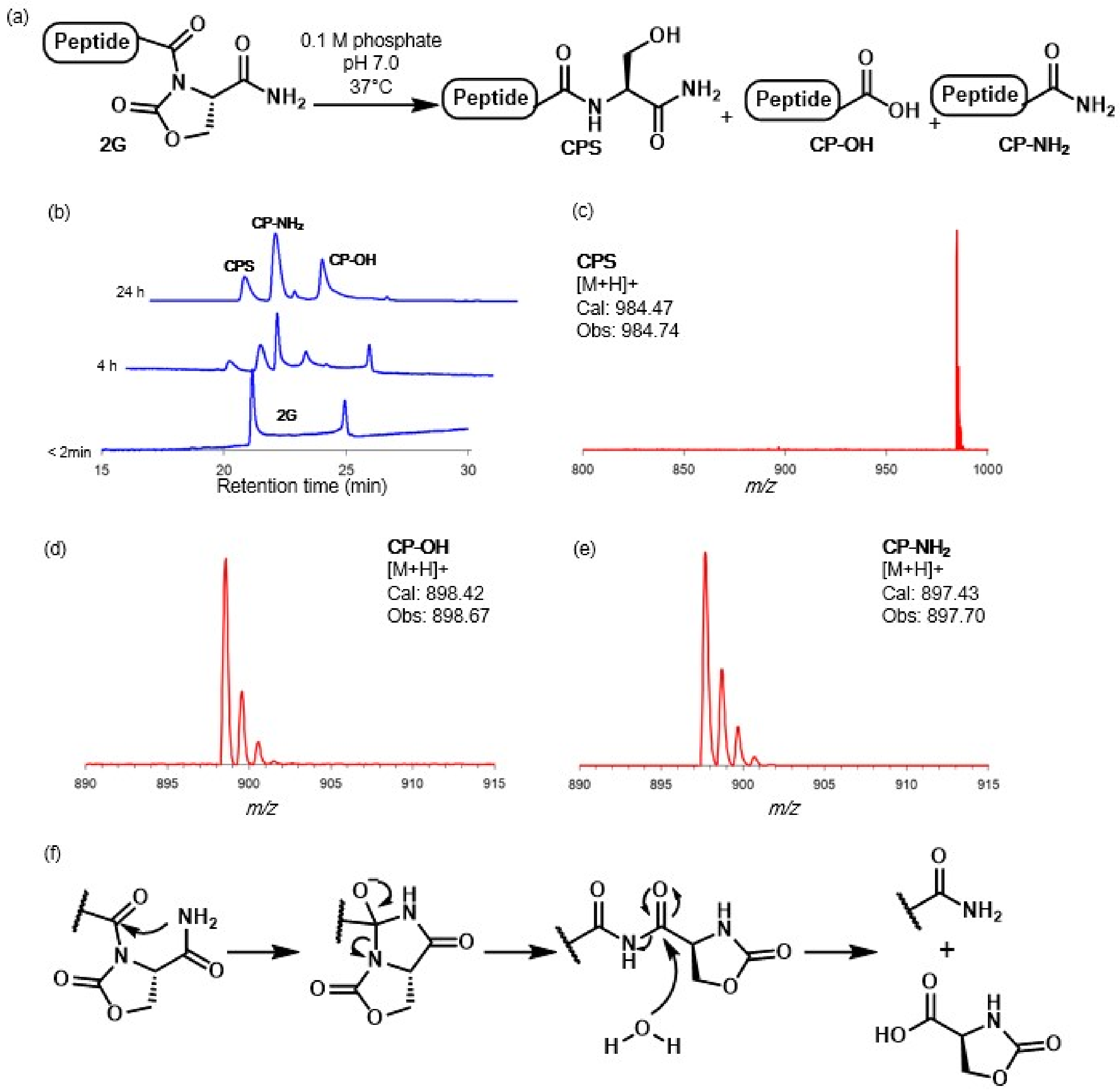

Figure S14. Stability assessment of CPOxd peptide **2G**. (a) Reaction scheme of this analysis. (b) HPLC trace of stability assessment of CPOxd peptide at each time point. MALDI-MS of hydrolyzed product peptide (c) **CPS**, (d) **CP-OH** and (e) **CP-NH$_2$**. (f) Plausible reaction mechanism to produce **CP-NH$_2$**.

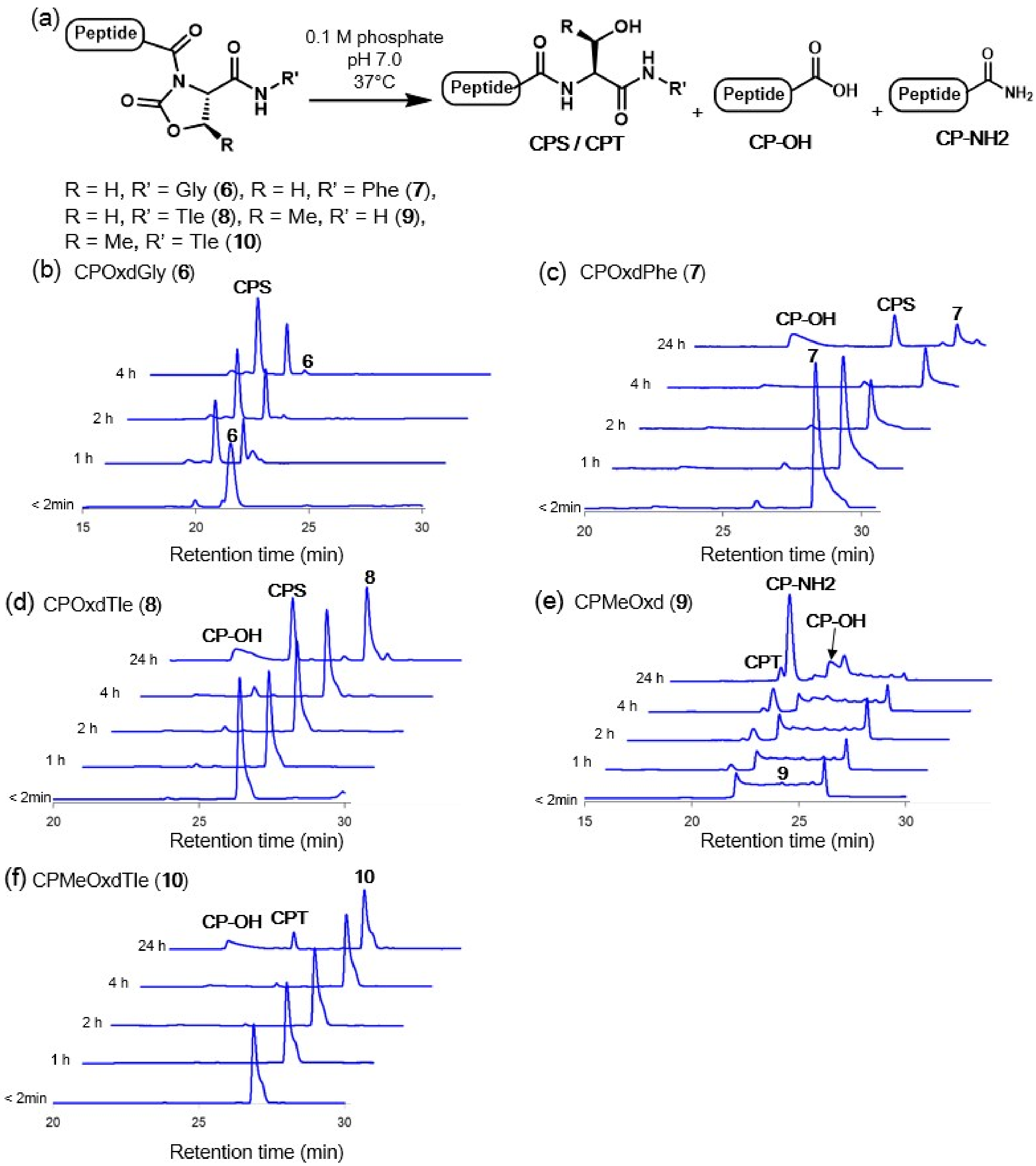

Figure S15. Stability assessment of CPOxd/CPMeOxd peptides (**6**-**10**). (a) Reaction scheme of this analysis. HPLC trace of stability assessment of (b) CPOxdGly (**6**), (c) CPOxdPhe (**7**), (d) CPOxdTle (**8**), (e) CPMeOxd (**9**) and (f) CPMeOxdTle (**10**) at each time point.

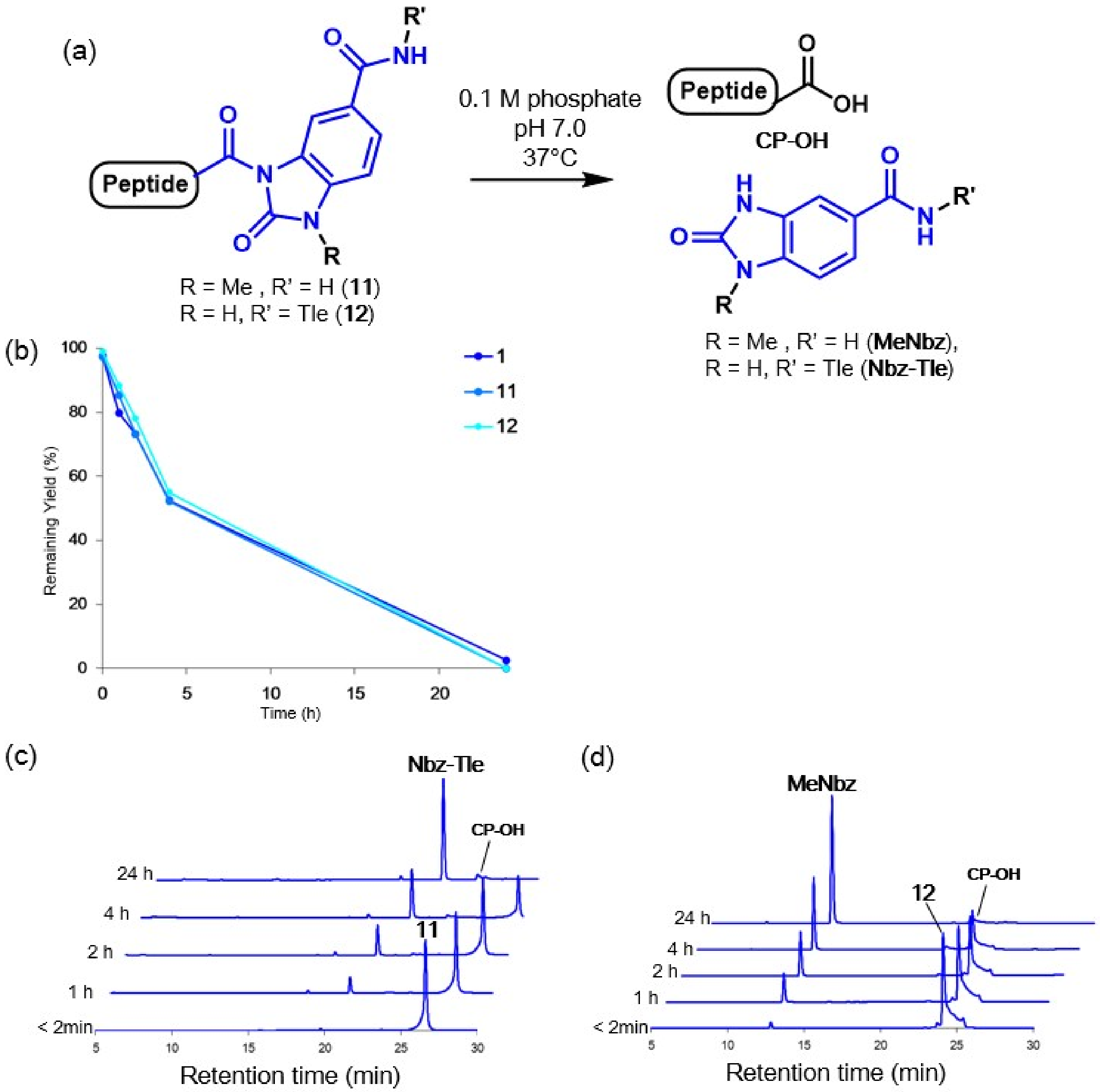

Figure S16. Stability assessment of peptides CPMeNbz-Tle (**11**) and CPMeNbz (**12**). (b) Time course profiles of CPNbz derivatives. (c) HPLC trace of stability assessment of **11** at each time point. (d) HPLC trace of stability assessment of **12** at each time point.

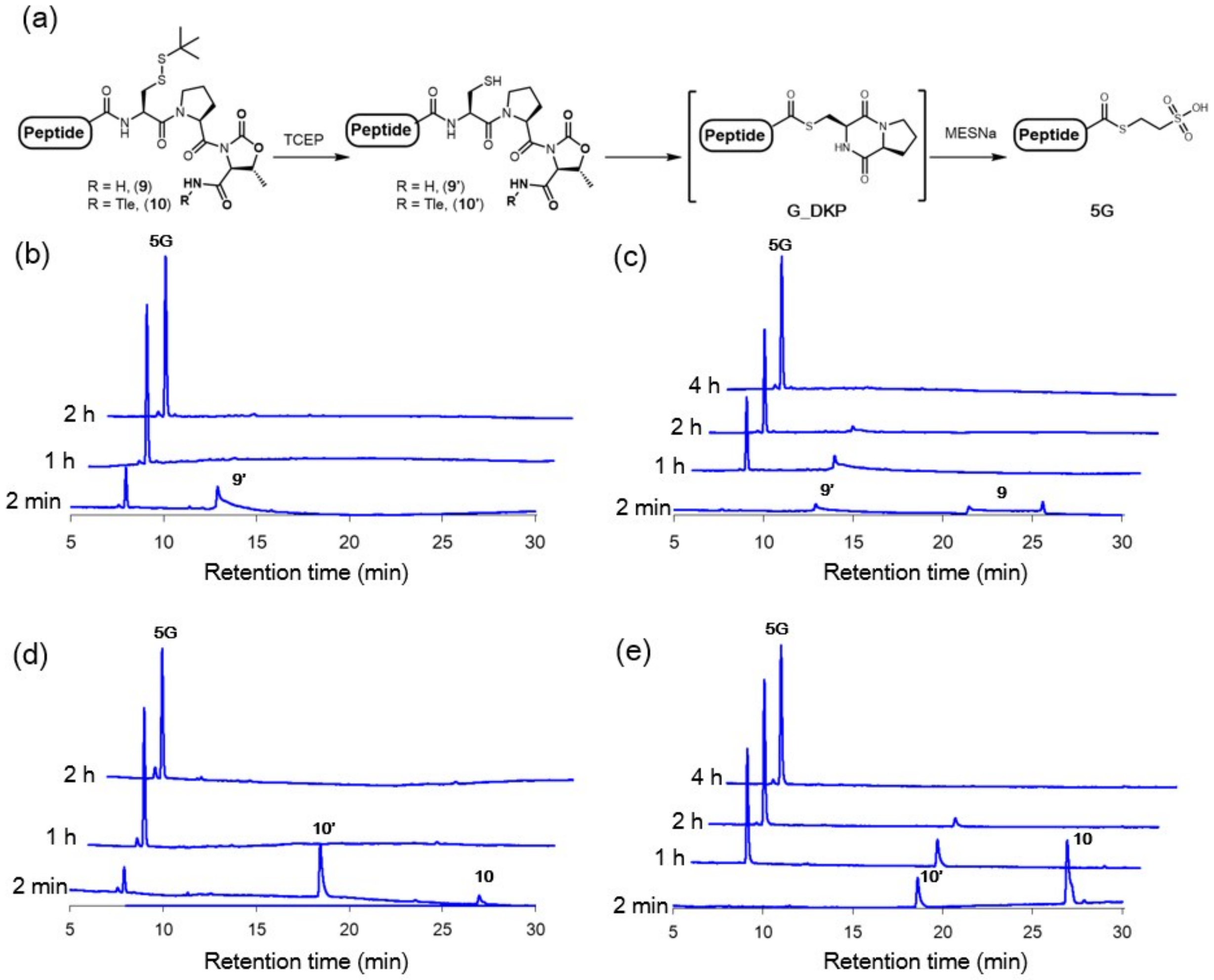

Figure S17. MESNa thioesterification of CPMeOxd peptides **9** and **10**. (a) Reaction scheme of this reaction. HPLC trace of MESNa thioesterification of peptide **9** at each time point at (b) pH 7.3 and (c) pH 6.0. HPLC trace of MESNa thioesterification of peptide **10** at each time point at (d) pH 7.3 and (e) pH 6.0.

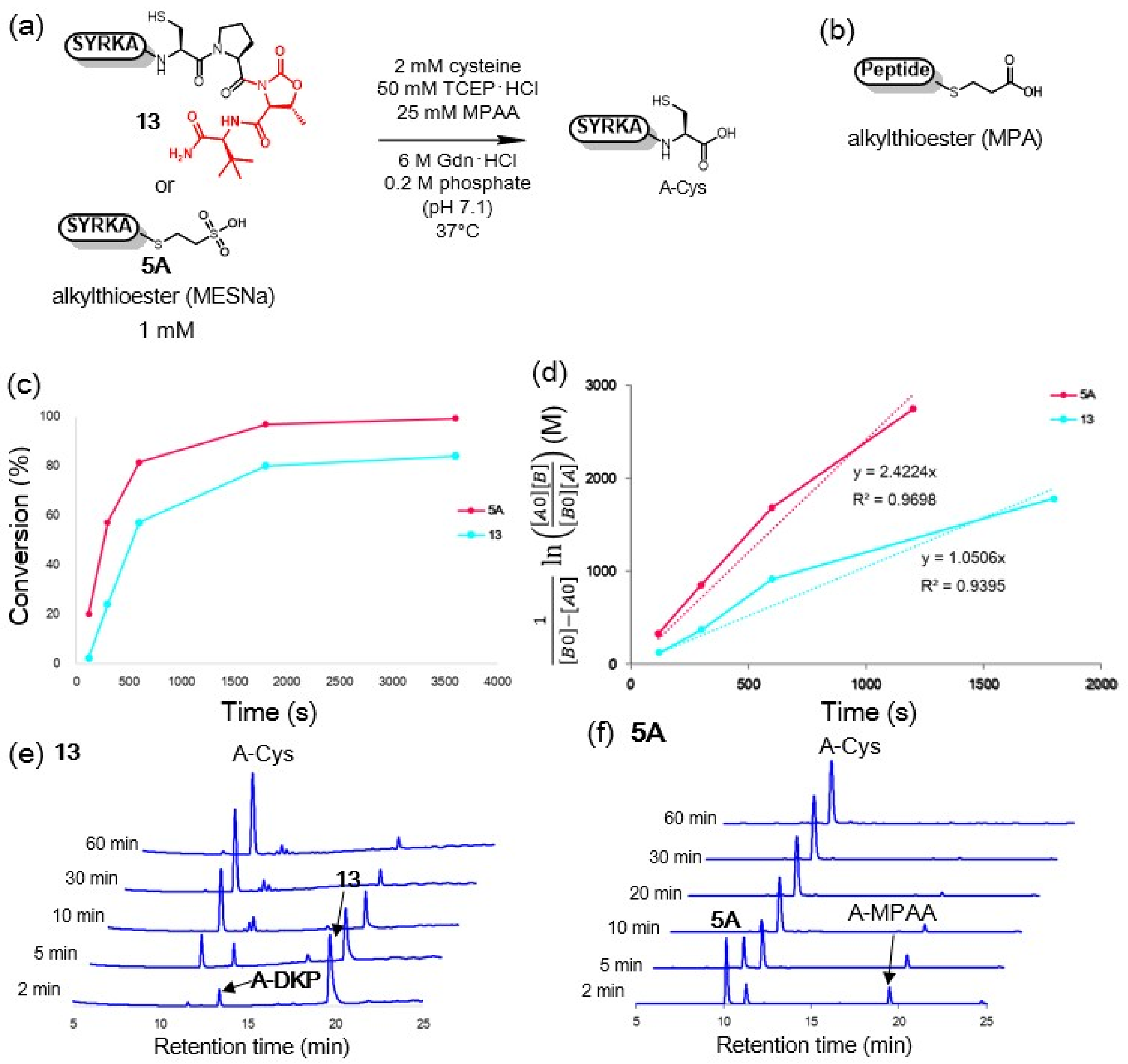

Figure S18. NCL kinetics analysis of peptides CPMeOxdTle **13** and alkylthioester (MESNa) **5A**. (a) Reaction scheme of this analysis. (b) Molecular structure of peptide alkylthioester (MPA) (c) Time course profiles of conversion yields. (d) Apparent second order kinetic constant for NCL using peptides **13** and **5A**. (e) HPLC trace of NCL using peptide **13** at each time point. (f) HPLC trace of NCL using peptide **5A** at each time point.

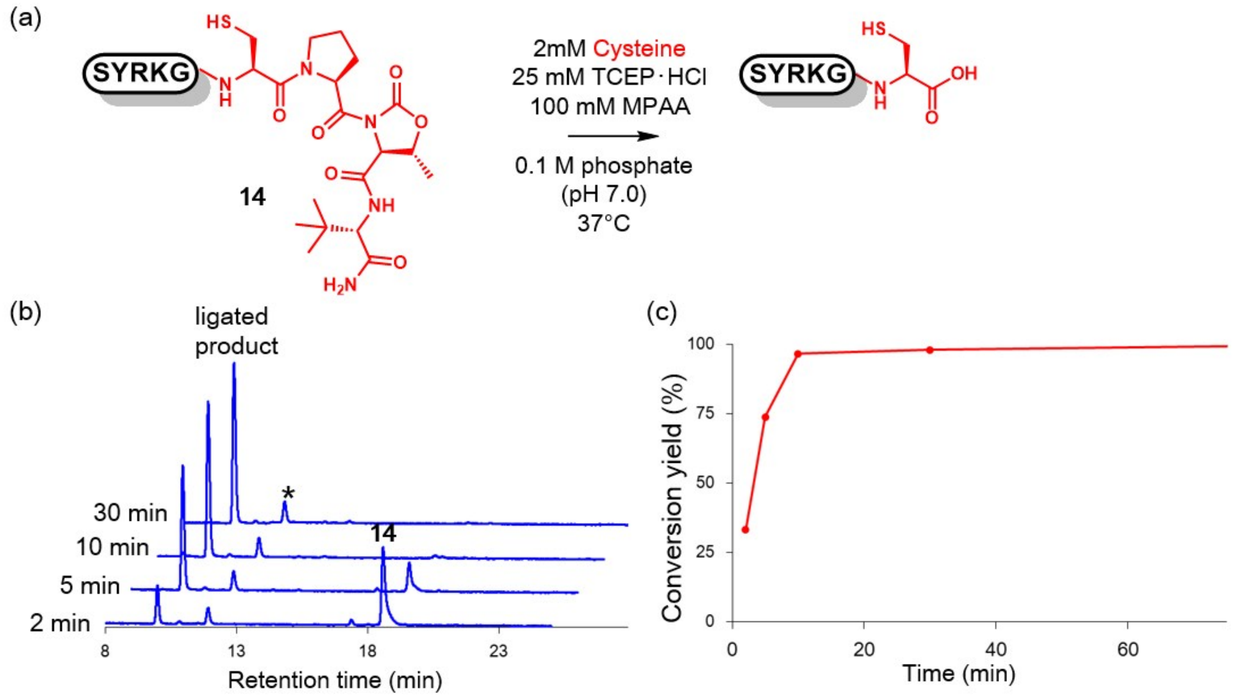

Figure S19. NCL kinetics analysis of peptide CPMeOxdTle (**14**). (a) Reaction scheme. (b) HPLC trace of NCL at each time point. * denote not-peptidyl peak. (c) Time course profiles of conversion yields.

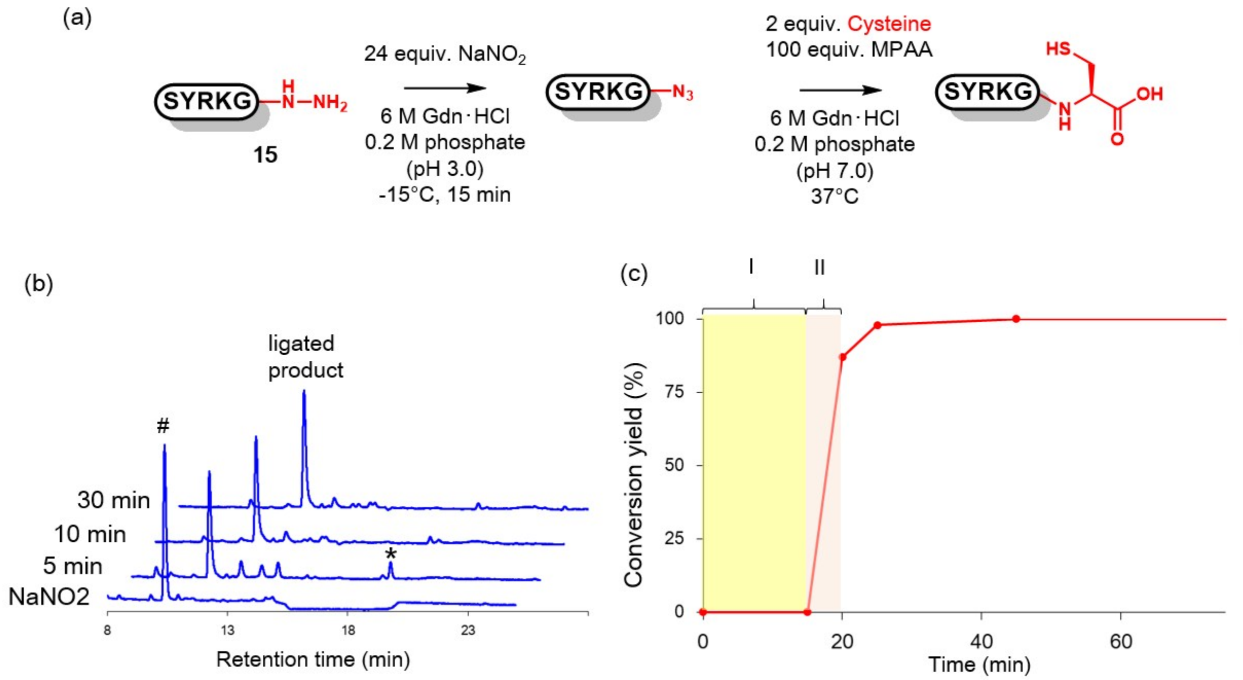

Figure S20. NCL kinetics analysis of peptide hydrazide (**15**). (a) Reaction scheme. (b) HPLC trace of NCL at each time point. # and * denote peptide azide and peptide MPAA thioester, respectively. (c) Time course profiles of conversion yields. (I): Azidation, (II): pH adjustment, temperature elevated.

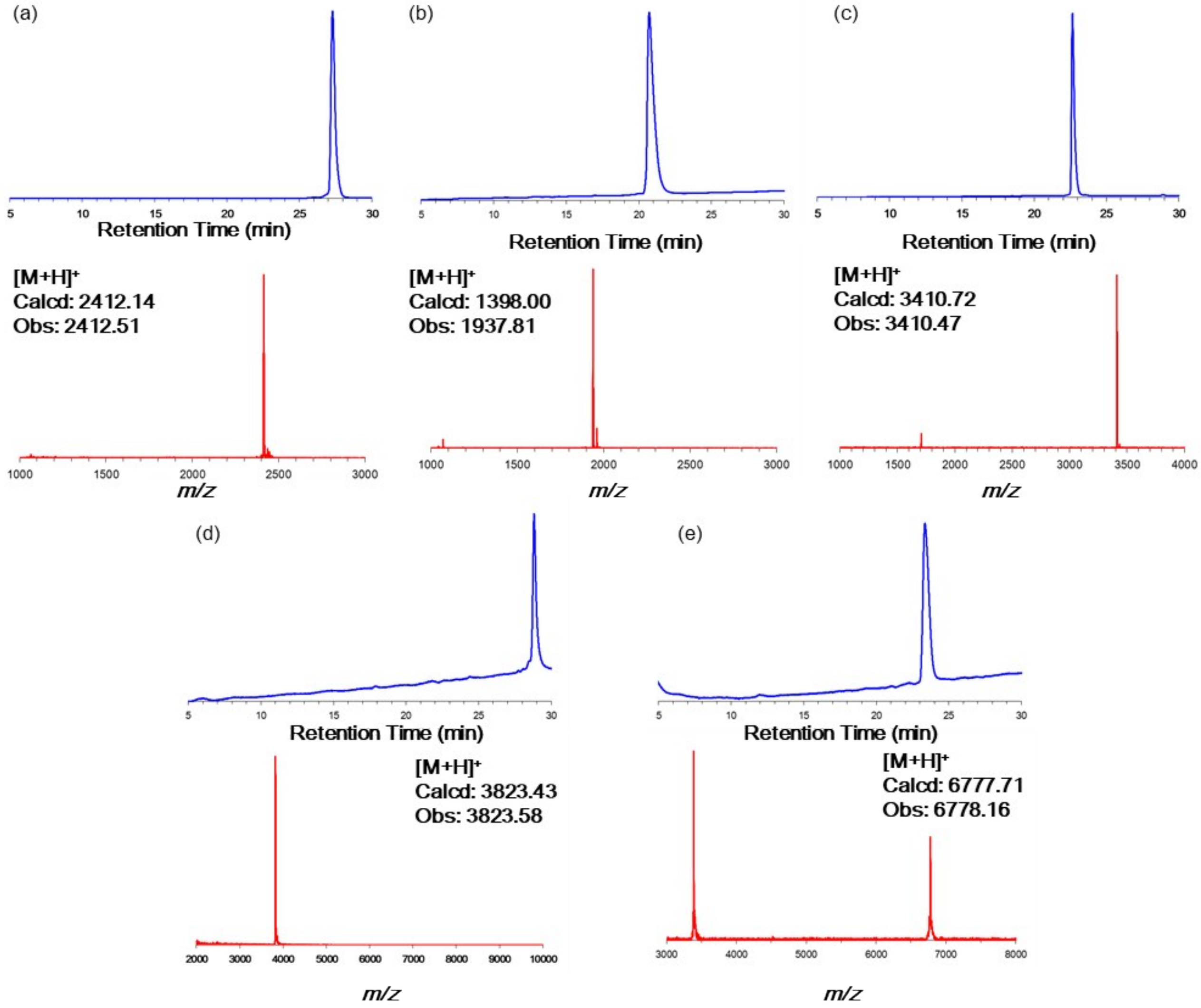

Figure S21. HPLC trace (blue) and MALDI-MS (red) of pure Affibody fragment (a) **Aff1**, (b) **Aff2**, (c) **Aff3**, (d) **Aff4** and (e) **Aff5**. Peptides were eluted from a Protein-R column (4.6×250 mm).

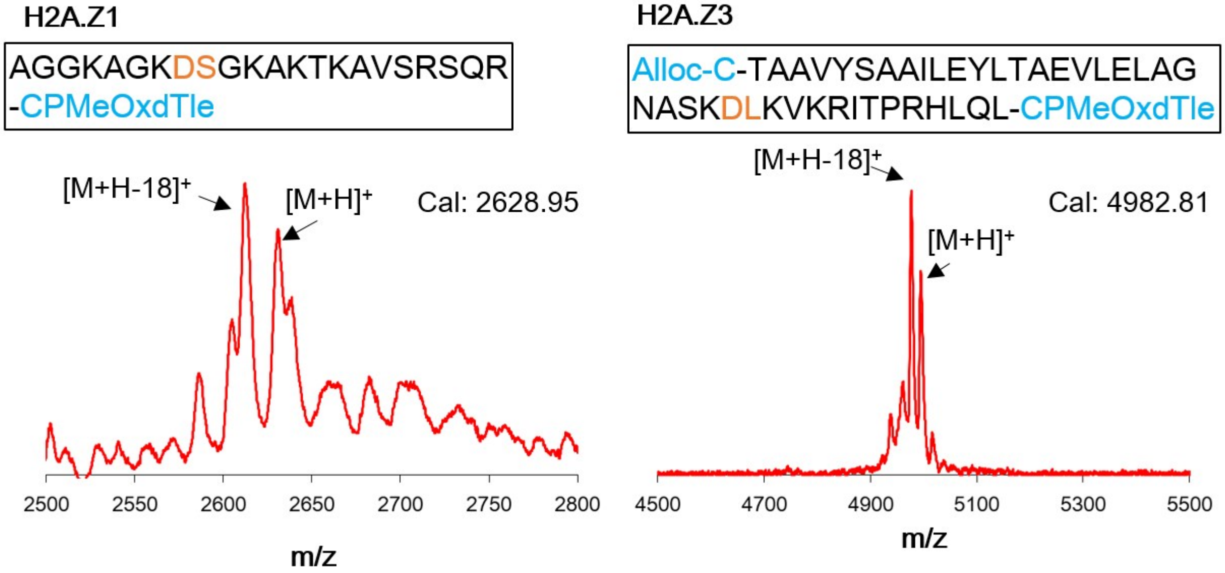

Figure S22. MALDI-MS of crude of initial synthesis of fragment **H2A.Z1** and **H2A.Z3**.

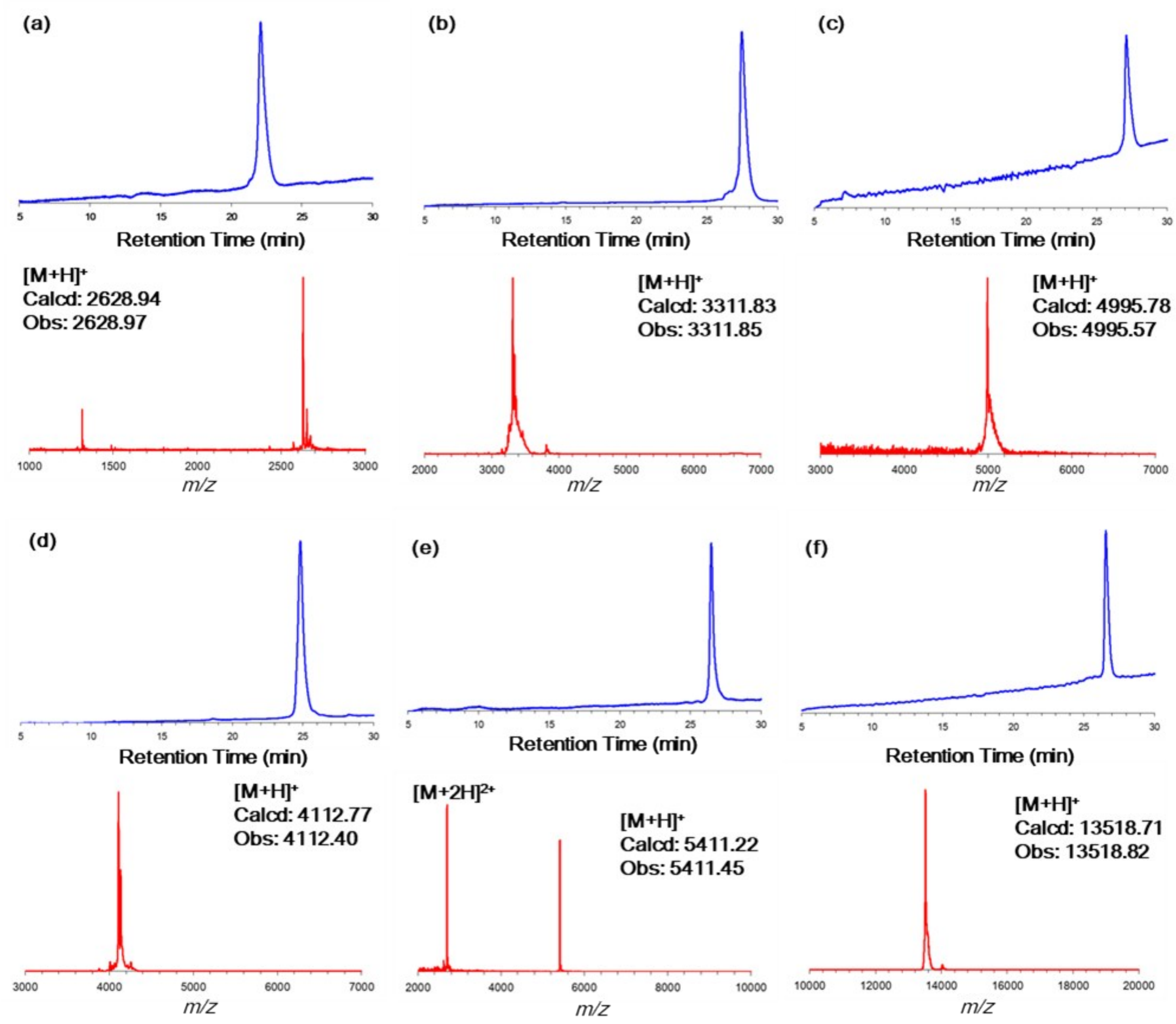

Figure S23. HPLC trace (blue) and MALDI-MS (red) of pure H2A.Z fragment (a) **H2A.Z1**, (b) **H2A.Z2**, (c) **H2A.Z3**, (d) **H2A.Z4**, (e) **H2A.Z5**, (f) **H2A.Z6**. Peptides were eluted from a Protein-R column (4.6×250 mm).

References

1. Huang, Y.; Li, Y.; Chen, Y.; Pan, M.; Li, Y.; Yu, L.; Guo, Q.; Liu, L. *Angew. Chem. Int. Ed.,* **2013***, 52*, 4858-4862.

2. (a) Kawakami, T.; Kamauchi, A.; Harada, E.; Aimoto, S. *Tetrahedron Lett.* **2014***, 55*, 79-81. (b) Asahina, Y.; Kawakami, T.; Hojo, H. *Chem. Commun.* **2017***, 53*, 2114-2117.

3. Zheng, J.; Tang, S.; Qi, Y.; Wang, Z.; Liu, L. *Nat. Protoc.* **2013***, 8*, 2483-2495.

4. (a) Kamo, N.; Hayashi, G.; Okamoto, *Chem. Commun.* **2018***, 54*, 43374340. (b) Kamo, N.; Hayashi, G.; Okamoto, *Angew. Chem. Int. Ed.* **2018***, 57*, 16533-16537.
